# Exosomes in Benign Urological Disorders: From Molecular Biology to Clinical Perspectives

**DOI:** 10.3390/ijms27146441

**Published:** 2026-07-20

**Authors:** Adelina Hrkać, Luka Bulić, Petar Brlek, Sven Nikles, Pero Bokarica, Tomislav Madžar, Dragan Primorac

**Affiliations:** 1 St. Catherine Specialty Hospital, 10000 Zagreb, Croatiapetar.brlek@svkatarina.hr (P.B.);; 2 University Hospital “Sveti duh”, 10000 Zagreb, Croatia; 3 International Center for Applied Biological Research, 10000 Zagreb, Croatia; 4 School of Medicine, Josip Juraj Strossmayer University of Osijek, 31000 Osijek, Croatia; 5 Faculty of Electrical Engineering and Computing, Algebra Bernays University, 10000 Zagreb, Croatia; 6 Department of Molecular Biology, Faculty of Science, University of Zagreb, 10000 Zagreb, Croatia; 7 Polyclinic “Vaš Pregled”, 10000 Zagreb, Croatia; 8 Faculty of Medicine, University of Applied Health Sciences, 10000 Zagreb, Croatia; 9 Faculty of Dental Medicine and Health, Josip Juraj Strossmayer University of Osijek, 31000 Osijek, Croatia; 10 Eberly College of Science, The Pennsylvania State University, State College, PA 16802, USA; 11 School of Medicine, University of Split, 21000 Split, Croatia; 12 The Henry C. Lee College of Criminal Justice and Forensic Sciences, University of New Haven, New Haven, CT 06516, USA; 13 Sana Kliniken Oberfranken, 96450 Coburg, Germany; 14 School of Medicine, University of Rijeka, 51000 Rijeka, Croatia; 15 School of Medicine, University of Mostar, 88000 Mostar, Bosnia and Herzegovina; 16 Department of Forensic Science, National Forensic Sciences University, Gandhinagar 382007, India; 17 School of Medicine, University of Pittsburgh, Pittsburgh, PA 15213, USA

**Keywords:** extracellular vesicles, exosomes, small extracellular vesicles, biomarkers, benign urological disorders, urology, bladder pain syndrome, erectile dysfunction, male infertility, regenerative medicine, microRNAs

## Abstract

Exosomes, nanoscale extracellular vesicles released by virtually all cell types, have emerged as pivotal mediators of intercellular communication and play a crucial role in the pathophysiology of numerous acute and chronic diseases, including a wide spectrum of urological disorders. By acting as sophisticated biological shuttles, exosomes transport a rich and highly specific molecular cargo—comprising proteins, lipids, messenger RNAs, microRNAs, and other nucleic acids—that reflects the physiological or pathological state of their cell of origin. Owing to these unique properties, exosomes are increasingly recognized as promising biomarkers for the diagnosis, prognosis, and monitoring of a broad range of inflammatory, degenerative, and neoplastic diseases. Beyond their diagnostic value, exosomes have attracted considerable attention as therapeutic tools, given their ability to promote tissue regeneration, modulate immune responses, and serve as potential targeted drug-delivery systems for small molecules, biologics, vaccines, and gene-based therapies. Notably, exosomes recapitulate many of the beneficial biological effects traditionally attributed to stem cells, while potentially offering a more practical alternative. As cell-free entities, they may reduce—though not entirely eliminate—several risks associated with cell transplantation, such as uncontrolled proliferation and immune rejection, and they raise fewer ethical concerns, making them attractive candidates for regenerative and precision medicine. In urology, the diagnostic, prognostic, and therapeutic applications of exosomes are rapidly expanding, with particularly promising advances observed in bladder, prostate, and kidney diseases. Growing evidence also supports their relevance in a variety of benign urological conditions, including erectile dysfunction, male infertility, neurogenic bladder, urethral stricture disease, stress urinary incontinence, and bladder pain syndrome. This review synthesizes contemporary knowledge on the biological significance and clinical potential of exosomes in urology, highlighting their emerging role as biomarkers and therapeutics, with a special focus on benign urological disorders. We emphasize that the current evidence base in benign urology is largely preclinical, and that clinical translation, although promising, remains at an early stage.

## 1. Introduction

Exosomes are nanosized extracellular vesicles enclosed in a bilayered lipid membrane, secreted by numerous cells, including stem cells, carrying cell-specific biomolecules. First mentioned by Trams et al. in 1981 [1], with a diameter of about 40–160 nm, exosomes contain DNA, mRNA, miRNA, long non-coding RNAs (lncRNAs), proteins, lipids, various cytokines, transcription factor receptors, and other bioactive substances. This molecular load is preserved from degradation by enzymes like RNases and proteases thanks to the lipid bilayer membrane envelope. Some of the protein components harbored by these extracellular vesicles are ubiquitous, including membrane transport and fusion-related proteins (like Rab, GTPases), heat shock proteins (like HSP70, HSP90), the four-transmembrane protein superfamily (like CD63, CD81), ESCRT complex-related proteins (like Tsg101, Alix), and integrins [2]. Other exosomal components are progenitor-cell-specific, which, according to the cell of origin, transmit anti-inflammatory, immunomodulatory, and tissue-regenerative impacts. Exosomes are representatives of their parental cells, behaving as intercellular messengers and couriers, harboring similar therapeutic effects to their cell of origin. The presence of proteomic elements and genetic materials indicates that exosomes can regulate and initiate certain signaling pathways, therefore modifying the transcriptional profile of the recipient cell [3,4]. Exosomes facilitate intercellular signal transduction via several mechanisms. For example, they carry biologically active molecules to activate or inhibit signaling pathways in recipient cells, transport receptors between donor and recipient cells to modify cellular activity, deliver fully functional proteins to execute specific tasks in target cells, and supply novel genetic information to recipient cells, which leads to the development of new phenotypes [5].

Unlike liposomes and other in vitro-synthesized nanocarriers, exosomes are endogenous vesicles and thus are expected to show greater biocompatibility and lower immunogenicity [2]. Exosomes are the exocytic product of practically any cell type, detectable in blood, saliva, urine, plasma, tears, semen, amniotic fluid, and even breast milk [6]. Once they reach the recipient cell, exosomes are further processed through processes such as membrane fusion, receptor interaction, and internalization [7]. Among cellular uptake routes, receptor-mediated endocytosis is a primary way in which exosomes interact with recipient tissues. Ligand–receptor recognition simplifies vesicle internalization, improving the uptake of the encapsulated payload and supporting efficient, relatively stable cargo transport. Exosomes also exhibit intrinsic tropism for certain cells. They can cross biological barriers, including the blood–brain barrier, which gives them a natural advantage as drug shuttles for the targeted delivery of nucleic acids and small-molecule therapeutics (from conventional medicines to plant-derived compounds). Native exosomes may show limited target specificity and can be cleared rapidly, reducing exposure and efficacy. To overcome this, they are often engineered, in other words, loaded, with therapeutic agents and/or surface-modified, to enhance targeting, stability, and pharmacokinetics [2].

Exosomes are gradually being tested as therapeutic agents across a wide range of conditions. For instance, in neurology, current papers report their use in Alzheimer’s and Parkinson’s disease, multiple sclerosis, traumatic brain injury, and peripheral nerve repair. They also display potential in modulating post-injury biology after acute organ damage (e.g., myocardial infarction, stroke) and in chronic degenerative settings such as diabetes and atherosclerosis. In regenerative medicine, exosome-based approaches are under investigation for bone and cartilage repair, osteoarthritis, cutaneous wound healing, hair follicle regeneration, intervertebral disc repair, spinal cord recovery, and vascular regeneration. Also, various studies are investigating exosomes as innovative vehicles for targeted cancer therapies due to their inherent activity and capacity for cargo transport [8]. Exosome-based therapies can be administered by multiple routes—intravenous, subcutaneous, intraperitoneal, intratumoral, intranasal, oral, and intravesical. The selected route strongly shapes therapeutic outcomes by determining biodistribution and in vivo clearance kinetics [2].

**Figure 1:**
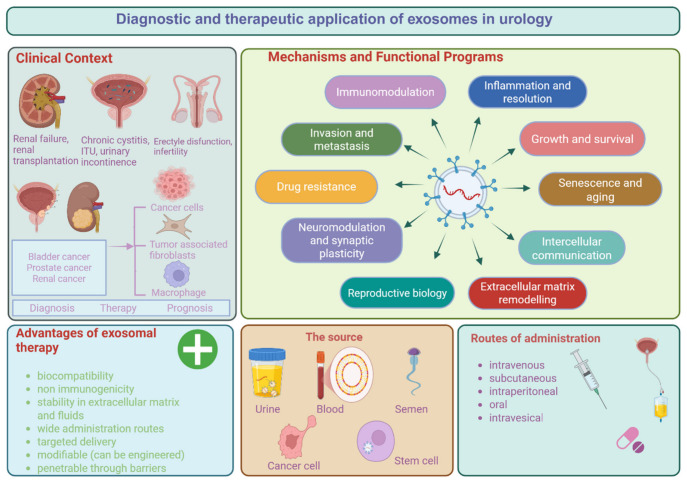
Overview of diagnostic and therapeutic applications of exosomes in urology. Clinical relevance: Exosomes play roles in renal and bladder cancer, chronic cystitis, urinary infections, incontinence, prostate disease, erectile dysfunction, infertility, renal failure, renal transplantation, and host–pathogen interactions. Biological mechanisms: They mediate immunomodulation, inflammatory regulation, cell growth and survival, invasion and metastasis, drug resistance, neuromodulation, reproductive signaling, aging processes, and ECM remodeling. Advantages: Exosomes offer biocompatibility, low immunogenicity, stability in biological fluids, targeted delivery potential, engineering flexibility, and capacity to cross biological barriers. Sources: Common urological sources include urine, blood, semen, cancer-derived cells, and stem cells. Delivery routes: Administration routes include intravenous, subcutaneous, intraperitoneal, oral, and intravesical delivery (Created in Biorender. Adelina Hrkać (2026)).

Several recent reviews, including articles published in this journal, have comprehensively covered the general biology, biogenesis, isolation, and characterization of extracellular vesicles, as well as their applications in oncology. The present review is intended to be complementary rather than duplicative: its distinct contribution is to consolidate the fragmented and predominantly preclinical evidence on extracellular vesicles across the spectrum of benign urological disorders within a single mechanistic framework. We deliberately focus on conditions for which disease-specific vesicle data have emerged—interstitial cystitis/bladder pain syndrome (IC/BPS), urinary tract infection, neurogenic bladder, urethral stricture, stress urinary incontinence, erectile dysfunction, male infertility, and Peyronie’s disease (Figure 1). Other benign urological conditions, such as benign prostatic hyperplasia, overactive bladder, chronic prostatitis/chronic pelvic pain syndrome, and urolithiasis, were considered beyond the scope of this review because dedicated vesicle research for these entities remains sparse; they are mentioned only where mechanistically relevant.

Stem cell therapy has been exploited in the field of regenerative medicine for a substantial period. Mesenchymal stem cells (MSCs) are the most frequently used, due to their potential for self-renewal and differentiation into descendant cells, for example, adipocytes or fibroblasts. However, despite their therapeutic promise, the clinical application of MSCs is limited by concerns related to tumorigenicity and immune activation, albeit these risks are relatively rare. Recently, the focus of academic society has shifted toward the mechanisms exploited in signaling pathways, which explain the therapeutic effect of MSC therapy. One of the possible vessels for transporting signaling molecules is exosomes, representing a compelling cell-free alternative characterized by better tolerance and superior effects compared to their cellular counterparts. Unlike their progenitor cells, MSC-derived exosomes have a favorable safety profile and can be more easily prepared and stored, thus overcoming logistical barriers. Some of the biological activities of MSC-derived exosomes include modulation of cell proliferation and migration, and apoptosis inhibition, as well as angiogenesis, neurogenesis, chondrogenesis, antifibrotic effects, and immunomodulation. Another ability of these vesicles is the creation of an anti-inflammatory microenvironment, which accelerates tissue repair and regeneration. Furthermore, exosomes isolated from MSCs offer a plethora of advantages over original stem cells, for example, optimal biocompatibility and isolation and storage protocols, as well as low antigenicity and oncogenic potential. These features put MSC-derived exosomes in the position of a safer and more adaptable, yet potent, alternative to traditional stem cell therapies in the management of diverse medical conditions. However, despite the therapeutic potential demonstrated in in vitro and animal studies, clinical trials concerning MSC-derived exosomes remain scarce. The reasons behind this disadvantage are the high production cost and uncertainty about the underlying mechanisms of action [2,3,9]. For instance, miRNAs can regulate the translation of several genes, some of which are outside our therapeutic framework, resulting in unexpected and unintended events. Additionally, the unstable and changeable metabolic state of the parental cells can modify exosome cargo. So, rigorous quality control is required to ensure safety and reproducibility at each step of exosome management (e.g., isolation, purification, characterization, modification, and storage). Overcoming these hurdles is essential before mesenchymal stromal cell-derived exosome (MSC-Exo) therapies can reliably move from bench to bedside [10]. The main differences between adult stem cell therapy and stem cell-derived exosome therapy are displayed in Table 1.

Exosomes are a part of the cancer biology network that act as couriers interlinking tumors to their microenvironment, while driving growth, immune escape, and metastatic spread. They transmit information that interferes with signaling pathways and reprogram the phenotype of recipient cells by carrying regulatory nucleic acids (miRNA, lncRNA, circRNA, mRNA) and nuclear or mitochondrial DNA alongside proteins and lipids [4]. Substantially, vesicle ligands and membrane proteins activate transmembrane receptors to initiate oncogenic cascades, while transferred small RNAs and lncRNAs remodel translation and transcription pathways. This results in an oncogenic “shift” that shapes the TME and seed dissemination [11]. During tumor progression, exosomes are identified as integral mediators of interaction between cancer cells and the surrounding microenvironment. The complex exosomal signaling interference between cancer cells and the TME influences crucial aspects of oncogenesis: invasion, angiogenesis, evasion of apoptosis, immune modulation, stromal remodeling, and ultimately metastatic spread [5]. The TME is not only an innocent observer in this set of events, but also an active participant, allowing cancer progression via the secretion of either activation or inhibitory molecules. This dynamic interaction mirrors the classical “seed and soil” hypothesis, accentuating the highly dynamic and reciprocal nature of tumor–stroma interactions and highlighting the crucial role of the TME in either promoting or inhibiting tumor evolution [12].

### Search Strategy and Scope

This article is a narrative (non-systematic) review. Relevant publications were identified through searches of PubMed/MEDLINE, Scopus, and Web of Science, covering the period from 2000 to 2025. Search terms combined “exosome(s),” “extracellular vesicle(s),” “small extracellular vesicle(s),” and “microRNA” with condition-specific terms. Priority was given to original preclinical and clinical studies, consensus guidelines, and recent peer-reviewed reviews published in English; reference lists were screened manually. Studies were included when they reported vesicle-related diagnostic or therapeutic findings relevant to benign urological disorders; non-peer-reviewed conference abstracts and non-English publications were excluded. As a narrative synthesis, the selection of studies was necessarily interpretive, and no formal quality or risk-of-bias appraisal was undertaken.

## 2. Classification

Extracellular vesicles integrate several subclasses, differentiated by their formation process, type of cargo, and characteristic sizes. Vesicles that bud directly from the plasma membrane, often termed ectosomes (including microvesicles or microparticles), typically measure roughly 50 nm to about 1 μm in diameter. By contrast, exosomes arise within the endosomal system (via multivesicular bodies) with generally lesser dimensions, on the order of 40–160 nm [13]. Detailed information is displayed in Table 2.

Exosomes are classified into two primary categories: natural and artificial. Natural exosomes can be categorized into groups according to their biological origin. Animal-derived exosomes comprise normal exosomes from healthy cells and tumor-derived exosomes, frequently carrying tumor-specific molecular cargo. Additionally, plant-derived extracellular vesicle-like nanoparticles constitute a distinct category of naturally occurring vesicles. Engineered exosomes are vesicles that are artificially modified, either functionally or physically, in order to enhance their efficacy in disease treatment, targeting, or diagnosis [2,11,14].

In line with the Minimal Information for Studies of Extracellular Vesicles (MISEV2023) guidelines [15], the generic term “extracellular vesicles” (EVs) is used throughout this review whenever the endosomal biogenesis of the vesicles under discussion has not been experimentally demonstrated, and “small EVs” (sEVs) denotes EVs smaller than approximately 200 nm. The term “exosome” is reserved for vesicles of confirmed endosomal (multivesicular-body) origin, and is otherwise retained only when reporting the terminology used by the authors of a cited study. Accordingly, several vesicle populations discussed here, including urinary, seminal, and plant-derived vesicles, as well as prostasomes and epididymosomes, are more precisely described as EVs or sEVs, and plant-derived vesicles are referred to as plant-derived EV-like nanoparticles.

## 3. Biogenesis and Utilization of Exosomes

**Figure 2:**
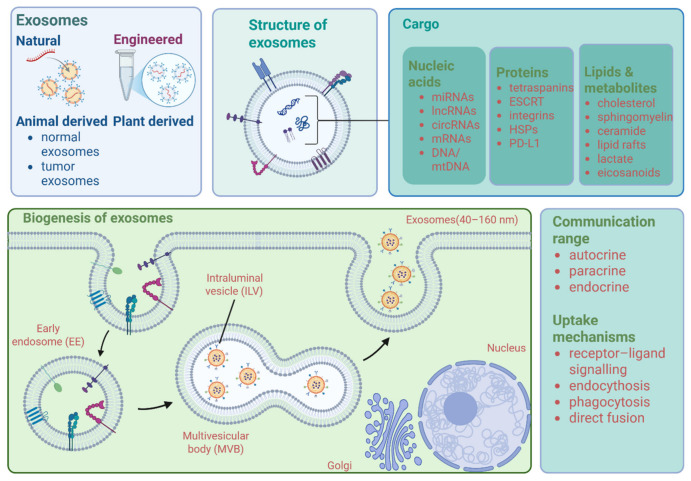
Overview of exosome types, structure, cargo composition, biogenesis pathways, and mechanisms of intercellular communication. Types of exosomes: Exosomes may be natural (from normal or tumor cells) or engineered, and can also originate from plant sources. Structure: Exosomes are 40–160 nm vesicles with a lipid bilayer containing transmembrane molecules, along with internal nucleic acids and metabolites. Cargo composition: They carry miRNAs, lncRNAs, circRNAs, mRNAs, DNA/mtDNA, proteins, and lipids. Biogenesis: Exosomes form through the endosomal pathway: early endosomes mature into multivesicular bodies, which release intraluminal vesicles as exosomes. Communication range: Exosomes mediate autocrine, paracrine, and endocrine signaling across short and long distances. Uptake mechanisms: Recipient cells internalize exosomes via receptor–ligand interactions, endocytosis, macropinocytosis, or direct membrane fusion (Created in Biorender. Adelina Hrkać (2026)).

The initial step in exosome formation is invagination of the cell membrane, followed by the collection of target bioactive substances, shaping early sorting endosomes (ESEs), followed by late sorting endosomes (LSEs), and finally multivesicular bodies (MVBs). MVBs then coalesce with the cell membrane, forming vesicles representing exosomes (Figure 2).

**Figure 3:**
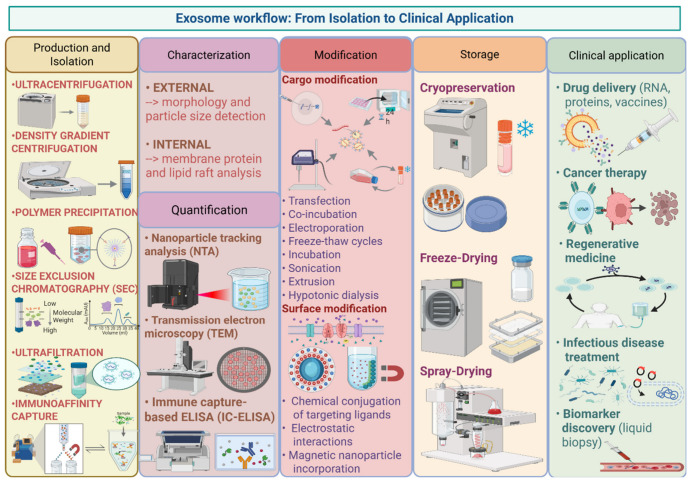
The figure summarizes key stages in the exosome utilization. Production and isolation rely on ultracentrifugation, density gradients, polymer precipitation, SEC, ultrafiltration, or immunoaffinity capture. Characterization includes assessment of morphology, size, membrane composition, and quantification using NTA, TEM, and IC-ELISA. Modification strategies involve cargo loading (e.g., transfection, electroporation, sonication) and surface engineering (chemical conjugation, electrostatic interactions, magnetic nanoparticle incorporation). Storage methods such as cryopreservation, freeze-drying, and spray-drying preserve exosome stability. Clinical applications span drug delivery, cancer therapy, regenerative medicine, infectious disease treatment, and biomarker discovery (Created in Biorender. Adelina Hrkać (2026)).

Preceding clinical use, exosomes have to be produced or isolated, depending on the determinant function and utility of the target exosomes. Produced exosomes can undergo additional modifications like drug loading or surface modification. Isolated exosomes must be purified and characterized. However, one of the exosomal characteristics is variation in size and content, which complicates their isolation. Additionally, the purity of the isolated material is compromised because the biochemical similarity of lipoproteins and other extracellular vesicles hinders the separation of exosomes. Therefore, efficient isolation of exosomes is currently a big issue, crucial for their further analysis. Various isolation techniques are employed based on the objective and sample type, including ultracentrifugation, density gradient centrifugation, polymer precipitation, size-exclusion chromatography (SEC), ultrafiltration, and immunoaffinity capture. The characteristics and comparative performance of the various exosome isolation techniques are summarized in Table 3 [2] (Figure 3).

After isolation, exosomes have to undergo characterization and quantification. According to the characterization goals and characteristics, two types of characterizations are exploited: external characterization, involving morphology and particle size detection, and internal characterization, such as membrane protein and lipid raft analysis. Currently available exosome characterization methods include nanoparticle tracking analysis (NTA), transmission electron microscopy (TEM) and immune capture-based ELISA (IC_ELISA). The main principles, advantages, and limitations of these methods are summarized in Table 4 [5,16].

Clinical rollout of exosome-based therapies is constrained by how little material current workflows produce. In routine lab settings, standard preparations often yield less than ~1 µg of exosomal protein per milliliter of conditioned medium, underscoring the efficiency bottleneck that must be overcome. To enhance exosome production, several strategies have been developed, for example, biochemical processes such as stimulation with LPS, BMP-2, HIF-1α, IFN-γ, and TNF-α, physical techniques including hypoxia, heat stress, and nutrient deprivation, mechanical methods like shear stress and 3D culture systems, and instrumental methods such as hollow-fiber bioreactors and stirred-tank bioreactors [3]. The positive aspects of potential exosome utilization are their ability to transport drugs, nucleic acids, and vaccines to specific target cells, while maintaining their stability and harboring minimal immunogenicity. They can also be biochemically modified to expand, alter, or enhance their therapeutic potential. Exosome modifications are primarily based on the modification target. Modifications of exosomal cargo include methods applied before exosome formation, such as transfection, co-incubation, electroporation, and procedures like freeze–thaw cycles, incubation, sonication, extrusion, and hypotonic dialysis, applied after production. Surface modifications are oriented to direct exosomes to the target cells. They are conducted on the membranes of the parental cells, involving chemical conjugation of targeting ligands, electrostatic interactions, and magnetic nanoparticle incorporation [3].

Exosomes have a major handicap in terms of limited stability over longer storage times. Appropriate handling can preserve their function and integrity. Exosome preservation methods, including cryopreservation, lyophilization (freeze-drying), spray-drying, and conventional storage at −80 °C, together with their principal advantages, limitations, and effects on exosomal stability and bioactivity, are summarized in Table 5 [3].

## 4. Application of Exosomes in Specific Urological Conditions

It should be emphasized at the outset that, apart from a limited number of human biomarker studies and early-phase trials, the great majority of the disease-specific data discussed below are derived from in vitro experiments and small-animal models. The strength of the underlying evidence therefore varies considerably among conditions; the level of evidence available for each disorder is summarized in Table 6.

### 4.1. Interstitial Cystitis/Chronic Bladder Pain Syndrome

Interstitial cystitis/bladder pain syndrome (IC/BPS) is a chronic condition characterized by a broad spectrum of lower urinary tract symptoms, including urinary frequency, urgency, nocturia, dysuria, and suprapubic or bladder-related pain that may radiate to other pelvic regions, in the absence of identifiable causes such as urinary tract infection, urolithiasis, or malignancy. The disease considerably impairs patients’ quality of life. The reported prevalence is highly variable, between 0.01% and 6.5%, with women being affected significantly more often than men. The precise etiopathogenesis remains incompletely understood. However, proposed mechanisms include chronic inflammation secondary to infection, autoimmune processes, mechanical injury, or other noxious stimuli, which comprise the processes leading to disruption of the glycosaminoglycan (GAG) layer, increased urothelial permeability, mast cell activation, neurogenic inflammation, neural and synaptic plasticity, and structural bladder wall changes such as fibrosis and loss of muscle fibers [16]. Persistent pain may lead to reflex pelvic floor muscle hyperactivity, further contributing to functional bladder outlet obstruction manifested by weak urinary stream, straining, and incomplete bladder emptying [17]. In addition to urinary symptoms, IC/BPS is commonly associated with systemic manifestations and comorbidities, including sleep disorders, chronic fatigue, sexual dysfunction, bowel disturbances and psychological distress such as anxiety. The primary goal of the treatment is symptom control and improvement of patients’ quality of life. Current guideline-based management is a multimodal and stepwise approach, which comprises behavioral and lifestyle modifications, oral pharmacotherapy, intravesical instillations and, in specific refractory cases, surgical intervention. However, therapeutic outcomes are still suboptimal in many patients, driving increased interest in novel treatment strategies, such as stem cell-based therapies, monoclonal antibody therapies and exosome-based approaches [16].

**Figure 4:**
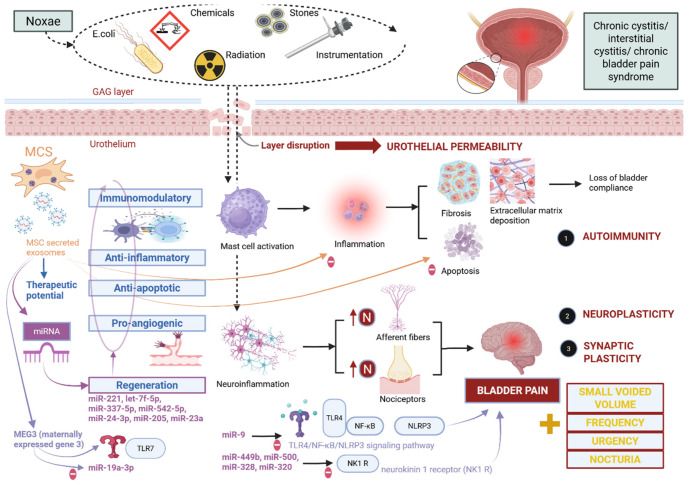
Schematic overview of the pathophysiological mechanisms underlying interstitial cystitis/bladder pain syndrome (IC/BPS). Environmental and iatrogenic noxae (infection, chemicals, radiation, and instrumentation) disrupt the urothelial glycosaminoglycan (GAG) layer, leading to increased urothelial permeability and mast cell activation. Subsequent inflammatory signaling promotes fibrosis and extracellular matrix deposition, resulting in reduced bladder compliance. Parallel neuroimmune interactions drive neuroinflammation, afferent nerve sensitization, and synaptic plasticity via NF-κB/NLRP3-dependent pathways, culminating in chronic bladder pain and lower urinary tract symptoms, including frequency, urgency, nocturia, and reduced voided volumes. The potential immunomodulatory, anti-inflammatory, and regenerative effects of mesenchymal stem cell-derived exosomes and miRNAs are highlighted as emerging therapeutic strategies (created with Biorender.com).

Mesenchymal stem cell (MSC)-derived exosomes have emerged as a promising therapeutic approach for IC/BPS due to their ability to modulate inflammation, promote tissue regeneration, and attenuate chronic pain signaling [18]. Experimental studies indicate that the beneficial effects of MSC-derived exosomes are mediated mainly through their bioactive cargo, which includes miRNAs, cytokines, and growth factors, collectively affecting multiple pathophysiological pathways involved in IC/BPS. Reported mechanisms include suppression of mast cell infiltration, reduction in inflammatory cytokine production, inhibition of apoptosis, modulation of extracellular matrix remodeling, and stimulation of urothelial repair and angiogenesis [19]. In parallel, MSC-derived exosomes appear capable of restoring bladder tissue homeostasis and improving structural and functional regeneration of the urinary bladder. Particular attention has been directed toward exosomal miRNAs, specifically fca-miR-221, fca-let-7f-5p, fca-miR-337-5p, fca-miR-542-5p, fca-miR-24-3p, fca-miR-205, and fca-miR-23a, which are increasingly recognized as important regulators of immune and neuronal signaling in IC/BPS [20]. Several studies demonstrated that exosome-associated miRNAs can suppress neuroinflammatory pathways, reduce bladder hypersensitivity, and modulate chronic pain transmission, for example, through regulation of TLR4/NF-κB/NLRP3-related signaling cascades and other neuromodulatory pathways [21]. Additional evidence suggests that prolonged inflammatory stimulation may alter the expression profile of specific exosomal miRNAs such as miR-449b, miR-500, miR-328, and miR-320, involved in pain adaptation and neurokinin 1 receptor desensitization, potentially representing an endogenous protective mechanism against chronic nociceptive overstimulation [22]. Exosomal cargo has been implicated in enhanced urothelial proliferation and tissue regeneration by releasing mitogenic and pro-angiogenic mediators such as basic fibroblast growth factor (bFGF) [23]. Molecules related to exosomes are being increasingly studied as potential diagnostic biomarkers in IC/BPS, in addition to their therapeutic potential. Altered expression patterns of some urinary exosomal non-coding RNAs and miRNAs have been identified in IC/BPS patients, and may be indicative of disease-related inflammatory and immune dysregulation. For example, MEG3 (maternally expressed gene 3) has been implicated in the pathogenesis of IC/BPS through suppressing miR-19a-3p expression and promoting TLR7 expression [24]. These results highlight the promise of urinary exosomal signatures as noninvasive biomarkers for disease diagnosis, phenotyping, and treatment response monitoring. In summary, growing evidence supports that MSC-derived exosomes have immunomodulatory, anti-inflammatory, anti-apoptotic, regenerative, and analgesic effects that may counteract some of the major mechanisms involved in the pathogenesis of IC/BPS. So, EV-based approaches represent an attractive cell-free treatment modality for IC/BPS. Still, further experimental and clinical studies are needed to determine the best sources and dosing strategies, long-term safety, and therapeutic efficacy of exosomes before widespread clinical application can be realized (Figure 4).

At present, the evidence in IC/BPS comprises exploratory human urinary biomarker studies (e.g., lncRNA MEG3 in Hunner-type disease) and therapeutic data confined to rodent and feline models; no vesicle-based therapy has yet been tested clinically in IC/BPS, and Hunner-type and non-Hunner disease should not be treated as interchangeable.

### 4.2. Urinary Tract Infections

Urinary tract infections (UTIs) are highly recurrent and prevalent infectious diseases. Clinical manifestations depend on the anatomical location of the infection. For example, lower urinary tract infections usually present with dysuria, urinary urgency and frequency, nocturia, and suprapubic discomfort without systemic manifestations. However, fever, chills, flank pain, or pelvic pain may suggest upper urinary tract involvement or prostatic infection, such as pyelonephritis or acute prostatitis. The clinical course is additionally influenced by numerous host-related and local predisposing factors. Susceptibility to infection is increased in advanced age, frailty, impaired immune function, pregnancy, urinary incontinence, atrophic vaginitis, and prostatic disease. Furthermore, poor bladder emptying due to anatomic, neurologic or functional abnormalities promotes urinary stasis and encourages bacterial colonization. Structural abnormalities such as urinary calculi or urinary tract obstruction also contribute to bacterial persistence, while catheterization and recent urologic interventions disrupt normal mucosal defenses and increase the risk of infection [25,26]. Antimicrobial therapy remains the treatment of choice, but the recurrence of infections and the increasing prevalence of multidrug-resistant pathogens represent major clinical challenges. Therefore, there is growing interest in other therapeutic approaches. Experimental evidence suggests that MSC-derived exosomes may display antimicrobial and immunomodulatory effects by inducing phagocytic activity, modulating inflammatory signaling and delivering antimicrobial molecules [27]. Proteomic analyses have identified innate immune proteins in human urinary exosomes with antibacterial activities able to inhibit the growth of Escherichia coli [28].

**Figure 5:**
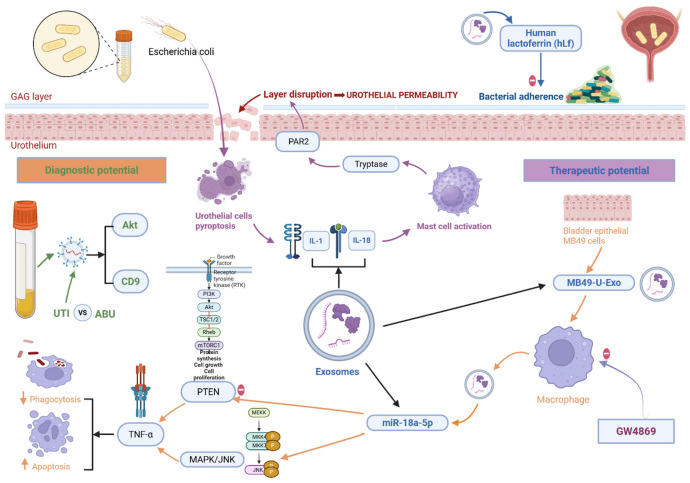
Proposed role of urothelial exosomes in urothelial barrier dysfunction and immune activation during urinary tract infection (UTI). Disruption of the glycosaminoglycan (GAG) layer and urothelial integrity by uropathogenic Escherichia coli increases urothelial permeability and facilitates bacterial adherence. Urothelial injury induces pyroptotic cell death and activates mast cells through protease-activated receptor 2 (PAR2) signaling, mediated in part by mast cell-derived tryptase. Mast cell activation amplifies the inflammatory cascade via exosomal interleukin-1 (IL-1) and interleukin-18 (IL-18) release. In parallel, urothelial cells secrete exosomes containing bioactive cargo, including miR-18a-5p, which modulates downstream inflammatory pathways. Exosomal miR-18a-5p suppresses PTEN and activates the MAPK/JNK signaling pathway, leading to enhanced TNF-α production, thereby decreasing phagocytosis and increasing apoptosis. The Akt/CD9 axis is highlighted as a potential diagnostic marker distinguishing symptomatic UTI from asymptomatic bacteriuria (ABU). Therapeutically, exosomes derived from MB49 bladder epithelial cells (MB49-U-Exo) demonstrate immunomodulatory effects on macrophages, while pharmacologic inhibition of exosome release with GW4869 attenuates these responses. Human lactoferrin (hLf) is depicted as an additional protective factor, reducing bacterial adherence to the urothelium. Collectively, the figure illustrates the dual diagnostic and therapeutic potential of urothelial exosomes in infection-driven bladder inflammation (created with Biorender.com).

Among uropathogens, uropathogenic Escherichia coli (UPEC) is recognized as the principal causative agent of UTIs and has therefore become the focus of extensive mechanistic research. Current evidence indicates that UPEC infection damages the urothelial barrier, exposing underlying bladder tissues to urinary components and inducing inflammatory cascades, including mast cell activation. One possible mechanism of action is UPEC infection-induced pyroptosis of urothelial cells, resulting in exosomal release of proinflammatory cytokines such as IL-1 and IL-18. These mediators induce mast cell activation with release of tryptase and activation of protease-activated receptor 2 (PAR2), further aggravating urothelial barrier dysfunction and favoring bacterial invasion [29]. Further studies showed that UPEC infection stimulated urothelial MB49 cells to secrete large amounts of exosomes enriched with inflammatory mediators and regulatory miRNAs. These miRNA-enriched exosomes may promote macrophage polarization toward a proinflammatory phenotype, enhanced TNF-α production, impaired phagocytic activity and apoptosis through PTEN and MAPK/JNK signaling pathways. Blockade of exosome release in experimental models has been associated with reduced bladder inflammation, indicating that exosome biogenesis and trafficking pathways may be novel therapeutic targets in UTI management [30]. Exosomes also seem to be involved in protective innate immune responses during UTIs. Urinary exosomes isolated from experimental models of UPEC infection were enriched in lactoferrin, an iron-binding glycoprotein secreted by bladder epithelial cells in response to infection. Lactoferrin administration in humans reduced bacterial adherence, increased neutrophil antimicrobial activity and decreased bacterial burden and inflammatory infiltration within the bladder, indicating a potential role of exosome-associated lactoferrin in host defense mechanisms [31]. Another clinically relevant challenge is differentiating symptomatic UTI from asymptomatic bacteriuria (ABU), especially in the elderly and institutionalized patients, where symptoms are often nonspecific. Since reliable diagnostic biomarkers remain lacking, recent investigations have focused on urinary exosomal signatures as potential discriminative tools. Altered expression of signaling molecules such as Akt, ERK, NF-κB, and exosomal surface proteins including CD9 has been observed in exosomes derived from infected urothelial and immune cells, suggesting that urinary exosomal profiles may provide a novel noninvasive approach for differentiating UTI from ABU [32] (Figure 5).

The roles attributed to vesicles in UTI differ in direction: UPEC-induced urothelial EVs may aggravate inflammation and barrier dysfunction, whereas other urinary vesicles may participate in host defense or serve as diagnostic markers. Most findings are derived from in vitro and animal models; the discrimination of UTI from asymptomatic bacteriuria, although promising, rests on small human samples and requires validation.

### 4.3. Neurogenic Bladder

Neurogenic bladder (NGB) is a frequent sequela of spinal cord injury (SCI), which carries a risk of vesicoureteral reflux (VUR) and subsequent chronic kidney damage. Conventional assessment, including urodynamics and urography, remains invasive and operationally complex. In this context, Li et al. [33] profiled urinary exosomes in SCI-related NGB. They found that vitronectin (VTN) was markedly enriched in patients with VUR versus those without it, indicating that urinary exosomal VTN may serve as a noninvasive biomarker for risk stratification, disease progression, and prognosis in NGB after SCI [33].

Current evidence in neurogenic bladder is limited to human urinary vesicle biomarker data (e.g., vitronectin as a predictor of vesicoureteral reflux); no therapeutic vesicle application has yet been reported for this condition.

### 4.4. Urethral Stricture

Urethral stricture is a benign urological condition that carries significant morbidity and a high risk of recurrence. The reported prevalence of urethral stricture is approximately 0.2–0.6% in men (roughly 229–627 per 100,000) [34]. In women, urethral stricture is comparatively rare and its epidemiology is poorly defined, with reported figures varying widely according to the population studied—for example, among women with bladder outflow obstruction, female urethral stricture has been reported in approximately 4–20% of cases [35]. The pathogenic process involves injury to or infection of the urethral wall, causing scarring of the urethral mucosa and underlying spongiosum tissue and subsequent constriction of the urethral diameter. The involved pathophysiological processes are fibroblast proliferation followed by excessive collagen deposition and significant chronic inflammation. Urethral strictures can be categorized based on their anatomical location (anterior versus posterior), cause (traumatic, inflammatory, idiopathic, iatrogenic), length (short-segment versus long-segment), and complexity (simple versus complex). Urethral stricture is clinically apparent through lower urinary tract symptoms on account of the bladder outlet obstruction. Diagnosis is obtained by clinical examination with thorough patient history, combined with retrograde urethrography or voiding cystourethrography and urethrocystoscopy. Treatment ranges from minimally invasive dilation and direct vision internal urethrotomy, which have high recurrence rates, to urethroplasty with superior long-term outcomes [34].

Emerging evidence suggests that potential exosome-mediated cross-talk between urothelial cells and neighboring mesothelial cells could be the central pathogenetic process in urethral fibrosis, which opens a window for some diagnostic and therapeutic possibilities. For example, macrophage-derived exosomes have been implicated in inflammation-related pathophysiologies, such as tissue injury and fibrosis repair. In line with this, Ren et al. [36] conducted research and showed that M2 macrophage-derived exosomes enriched in miR-34a-5p exacerbate fibrosis by switching off SIRT1 and blocking autophagosome–lysosome fusion in urethral fibroblasts, resulting in impaired fibroblast autophagy. Inhibiting miR-34a-5p mitigates urethral scarring, supporting the use of engineered exosomes to neutralize this microRNA as a potential treatment [36]. An animal study conducted by Liang et al. [37] elucidated the potential mechanism of fibrosis reduction mediated by adipose-derived stem cell exosomes. Applied exosomal content resulted in the suppression of the TGF-β/Smad and downstream PDGFR-β/RAS/ERK pathways, subsequently reducing collagen deposition and improving the uroflow pattern in a urodynamics study [37]. Wang and his associates went a step further. They produced a collagen/poly(L-lactide-co-caprolactone) (P(LLA-CL)) nanoyarn scaffold enriched with adipose-derived stem cell exosomes and incorporated it into the damaged urethra. As a result, enhanced epithelialization, vascularization, and anti-inflammatory responses were documented, with no evidence of scar formation. These findings offer promising regenerative potential for clinical urethral defect repair [38]. Another study experimenting on New Zealand rabbits showed a significant reduction in urethral stricture following the injection of Exo-MSCsIL-1β. These IL-1β-stimulated MSC-derived exosomes contain let-7c, which targets the PAK1-NF-κB signaling pathway and results in promotion of M2 macrophage polarization and inhibition of fibroblast activation. Thus, Exo-MSCs^IL-1β enriched with let-7c show promising therapeutic potential for urethral stricture treatment [39]. Further experiments by the same set of authors demonstrated that M2 macrophages secreted exosomal miR-381, which inhibits YAP/GLS1-dependent glutaminolysis in urethral fibroblasts. This chemical reaction is one of the initiating steps in the activation of urethral fibroblasts; thus, inhibition of this process results in a reduction in myofibroblast transformation and fibrosis [40]. Bone marrow MSC-derived exosomes also prevented urethral stricture in a rat model by downregulating fibrosis-related genes, specifically Col I, fibronectin, and elastin, and upregulating VEGF, eNOS, and bFGF, resulting in amplified angiogenesis [41]. Similarly, TNF-α-preconditioned MSC-derived exosomes derived from human umbilical cord also effectively suppressed urethral fibrosis and stricture. This antiscarring effect is due to the overexpression of an exosomal miR-146a in TNF-α-stimulated MSCs, resulting in the suppression of fibroblast activation [42]. Detailed information about exosomal effects on urethral stricture is displayed in Table 7.

It should be noted that vesicles exert opposing effects in this setting: M2 macrophage-derived vesicles enriched in miR-34a-5p can promote fibrosis, whereas stem cell-derived vesicles are predominantly antifibrotic. Their net effect is therefore context-dependent, and all of the data summarized here derive from preclinical models.

**Figure 6:**
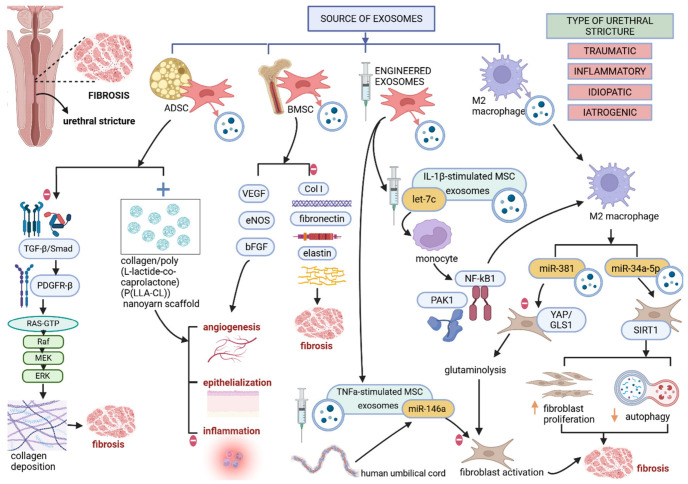
Exosome-mediated regulation of fibrosis and tissue remodeling in urethral stricture disease. This schematic summarizes the molecular pathways involved in urethral stricture-associated fibrosis and the modulatory effects of native and engineered exosomes. Stricture formation, arising from traumatic, inflammatory, idiopathic, or iatrogenic causes, is driven by activation of TGF-β/Smad and PDGFR-β–RAS–ERK signaling, leading to fibroblast activation and collagen deposition. Exosomes derived from adipose- and bone marrow-derived stem cells promote angiogenesis and tissue repair through pro-regenerative factors, while influencing extracellular matrix components. Engineered MSC-derived exosomes, including IL-1β-stimulated exosomes enriched in let-7c and TNF-α-stimulated exosomes carrying miR-146a, attenuate inflammatory and profibrotic signaling via inhibition of NF-κB and PAK1 pathways. The figure also highlights the contribution of M2-polarized macrophages, whose exosomes containing miR-381 and miR-34a-5p promote fibroblast activation through YAP/GLS1-dependent glutaminolysis and SIRT1 signaling, thereby sustaining fibrosis. Overall, the diagram illustrates how exosome-mediated intercellular communication may either drive or therapeutically mitigate fibrotic remodeling in urethral stricture disease (created with Biorender.com).

To conclude, these studies offer a possibility of exploitation of exosome-based therapies as a promising strategy for the prevention and treatment of urethral stricture, owing to the exosomal antiscarring properties exploited through targeting of key fibrogenic pathways (Figure 6).

### 4.5. Stress Urinary Incontinence

Stress urinary incontinence (SUI) is a common urological disorder affecting 11–70% of the population, with prevalence increasing with growing age. It is characterized by the involuntary leakage of urine when bladder pressure exceeds urethral closing pressure during routine activities, such as physical exertion, coughing, exercise, or sneezing, which increases intra-abdominal pressure. The underlying pathophysiological mechanism is either urethral hypermobility caused by impaired supporting structures or intrinsic urethral sphincter deficiency, characterized by deterioration of the urethral mucosa and diminution of the urethral sphincter muscle tone. These structural changes typically occur with aging, but are also more frequent after pregnancy and childbirth, or following pelvic operations or radiotherapy. Standard therapeutic options for SUI include conservative and surgical treatments, each with some advantages and disadvantages. For example, a conservative approach is associated with high recurrence rates, partially due to poor patient compliance. In contrast, surgical interventions, such as sling procedures, require a good performance status and carry a risk of surgical and postsurgical complications, including mesh exposure due to foreign body reactions and poor localized tissue healing. Conservative treatment includes adjustment of life habits involving diet modification, exercise, and pelvic floor muscle training with or without electrostimulation or functional magnetic stimulation. Surgical treatment includes sling operations, utilizing polypropylene mesh and colposuspension, which focus on re-establishing suburethral support [43]. The above-mentioned therapy limitations emphasize the need for treatment modalities that can achieve a satisfactory success rate while employing a minimally invasive approach.

Exosomes present a promising alternative to conventional SUI therapies thanks to their regenerative properties and the potential to repair nerve and muscle damage and restore urethral stability. Also, exosomes can be applied as a minimally invasive therapy, thereby reducing the risk of complications associated with current procedures. Studies concerning the utilization of exosomes in SUI treatment are mainly based on the regenerative properties of stem cells and their exosomal derivatives. For example, Li et al. [44] evaluated the therapeutic effect of exosomes (SIRT1/exos) derived from SIRT1-overexpressing human bone marrow mesenchymal stem cells (BMSCs) in a stress urinary incontinence (SUI) rat model. Exosomes activated the ERK pathway in satellite cells, causing their proliferation and differentiation, and ultimately improving leak point pressure and bladder capacity in vaginal dilation-induced SUI models [44]. Another rat study evaluated the therapeutic efficacy of adipose tissue-derived stem cell exosomes (ADSCEs) in SUI models, demonstrating that systemic administration of ADSCEs preserved urethral sphincter thickness (*p* < 0.01), reduced fibrosis and collagen deposition, and promoted tissue remodeling, resulting in an increase in abdominal leak point pressure (ALPP) and reducing the symptoms of SUI [45]. Ni and colleagues tested human adipose-derived stem cell (hADSC) exosomes as a regenerative therapy for stress urinary incontinence. Proteomic and pathway analyses showed that these vesicles are enriched in mediators of PI3K–Akt, JAK–STAT, and Wnt signaling, networks tied to skeletal muscle and peripheral nerve growth. In vitro, they drove dose-dependent proliferation of myogenic cells and Schwann cells. In a rat SUI model, intraparenchymal delivery of hADSC exosomes improved bladder capacity and leak point pressure, while histology demonstrated greater striated muscle content and denser peripheral nerve fibers versus untreated controls. Together, the data position hADSC exosomes as a plausible, cell-free candidate for functional and structural restoration in SUI [46]. According to Liu and his associates, adipose-derived stem cell (ADSC) exosomes have been shown to upregulate type I collagen by increasing col1a1 expression while suppressing collagen-degrading enzymes by downregulating MMP-1 and MMP-2 expression in periurethral fibroblasts from women with SUI [47]. Urine-derived stem cells (USCs-Exo) serve as a potential therapeutic strategy for SUI treatment. According to Wu and his colleagues, USC-derived exosomes enhanced the activation, proliferation, and differentiation of rat muscle satellite cells (SCs) by promoting the phosphorylation of extracellular-regulated protein kinases (ERKs), which triggers muscle regeneration, opening the possibility for further exploration in SUI treatment [48]. Rolland and his associates experimented with in vitro and in vivo SUI models, employing a human platelet-derived extracellular vesicle product (PEP) that expressed CD41a and CD9 and was enriched in NF-κB and PD-L1, designed for sustained delivery via hydrogels. In vitro, PEP promoted proliferation and differentiation of skeletal muscle satellite cells through NF-κB signaling. In an animal model, PEP-enhanced hydrogel stimulated skeletal muscle regeneration, leading to the restoration of external urethral sphincter function, and promoted regenerative M2 macrophage polarization [49]. M2 macrophages are very important mediators of posttraumatic myoblast differentiation. M2 macrophage-derived exosomes (M2-EXO), enriched in miR-501, were exploited in the in vivo SUI model study of Zhou et al. [50]. The results demonstrated extensive M2 macrophage infiltration at the pubococcygeal muscle injury site by day 5 post injury, significantly enhanced myoblast differentiation into myotubes, reduced inflammation, and improved recovery of damaged pubococcygeal muscle, highlighting exosomal miR-501 as a promising therapeutic candidate for muscle injury-associated conditions such as SUI [50].

**Figure 7:**
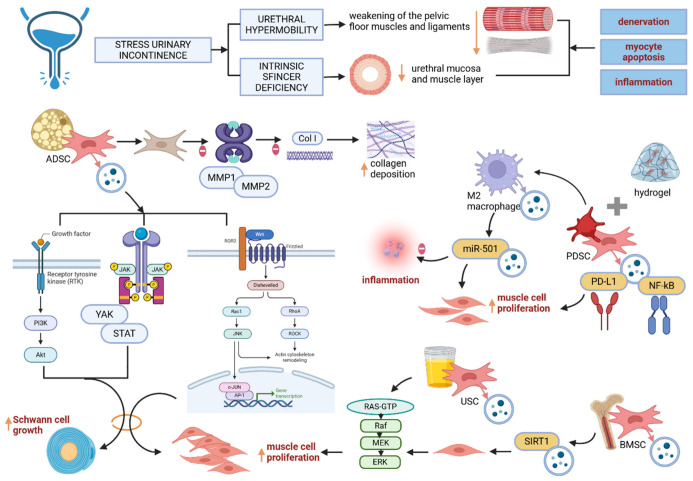
Pathophysiology of stress urinary incontinence (SUI) and regenerative mechanisms of stromal cell exosome-based therapies. The figure summarizes the multifactorial pathogenesis of SUI, driven by urethral hypermobility due to pelvic floor weakening and intrinsic sphincter deficiency characterized by loss of urethral mucosa and muscle. These processes are associated with denervation, myocyte apoptosis, and chronic inflammation, resulting in impaired urethral closure. The lower panels depict regenerative and immunomodulatory pathways mediated by adipose-derived, periurethral/perivascular, urine-derived, and bone marrow-derived stem/stromal cells and their extracellular vesicles. These cells promote extracellular matrix remodeling (via MMP1/2 and collagen I regulation), muscle and Schwann cell proliferation (PI3K/Akt, JAK/STAT, Wnt, and ERK pathways), and immune modulation through M2 macrophage polarization, microRNA signaling (e.g., miR-501), NF-κB inhibition, PD-L1 signaling, and SIRT1 activation. Collectively, these mechanisms support muscle regeneration, neural repair, and inflammation reduction, offering a rationale for regenerative therapies in SUI (created with Biorender.com).

Taken together, these findings expand current understanding of regenerative mechanisms. Exosome-based therapy, from hydrogel systems (PEP) and engineered MSC-Exos (SIRT1) to endogenously derived macrophage or USC exosomes, may outcompete invasive or short-lived therapies for SUI. Detailed information regarding the usage of exosomes in incontinence pathology is displayed in Table 8 and Figure 7.

The regenerative effects reported in stress urinary incontinence are consistent but derive exclusively from preclinical models (rodent and porcine) and in vitro work; clinical confirmation is lacking.

### 4.6. Erectile Dysfunction

Erectile dysfunction (ED) is a highly prevalent urological condition affecting over 50% of men above the age of 50, frequently driven by underlying vasculogenic, neurogenic, or psychogenic factors or a combination of these. While conventional treatments ranging from peroral phosphodiesterase-5 inhibitors to penile prosthesis implantation remain the standard of care, many patients experience suboptimal outcomes. Consequently, exosomes have emerged as a highly promising, cell-free regenerative therapy capable of addressing the root pathophysiological causes of ED. Preclinical evidence highlights their efficacy across several distinct ED etiologies [51,52].

#### 4.6.1. Diabetic Erectile Dysfunction

In diabetic ED, the pathophysiological core involves severe endothelial dysfunction, accelerated apoptosis, and cavernous fibrosis. Exosome therapies in these models consistently demonstrate antifibrotic and pro-angiogenic mechanisms. For instance, NO/cGMP pathway restoration and fibrosis reduction have been achieved using exosomes derived directly from corpus cavernosum smooth muscle cells (CCSMCs) [51]. Adipose-derived stem cell (ADSC) exosomes significantly improve erectile function by increasing the smooth muscle-to-collagen ratio, enhancing endothelial marker expression (CD31) and reducing apoptosis via Bcl-2 upregulation [52]. These regenerative effects are largely dictated by the exosomal cargo. ADSC-derived exosomes loaded with pro-angiogenic (miR-126, miR-130a, miR-132) and antifibrotic (miR-let7b, miR-let7c) microRNAs actively promote endothelial cell proliferation and reduce cavernous fibrosis [53]. Furthermore, modifying ADSC exosomes to carry specific proteins, such as corin, elevates beneficial proteins (ANP, BNP, nNOS) and curtails penile inflammation, directly improving the intracavernosal-pressure-to-mean-arterial-pressure (ICP/MAP) ratio [54].

#### 4.6.2. Vasculogenic, Hypoxic, and Age-Related Erectile Dysfunction

Exosomes also show therapeutic potential for reversing structural degradation tied to vascular injury, aging, and chronic hypoxia (such as obstructive sleep apnea). In age-related ED, MSC-derived exosomes successfully inhibit cellular apoptosis and restore erectile mechanics, operating primarily through the delivery of miR-296-5p and miR-337-3p to regulate the PTEN/PI3K/AKT signaling pathway [55]. In cases of hypoxia-induced ED, exosomal delivery of miR-301a-3p or circPIP5K1C reprograms smooth muscle cells toward a contractile, survival-favored phenotype by inhibiting glycolysis, augmenting autophagy, and suppressing apoptosis through the PTEN/HIF-1α and TLR4 axes [56,57]. Finally, in models of internal iliac artery injury, MSC-derived exosomes hasten endothelial repair, blunt oxidative stress, and restore nitric oxide synthase levels [58]. When engineered to overexpress miR-126, these vesicles robustly drive neovascularization. Zou and colleagues used muscle-derived stem cells engineered to overexpress microRNA-126. Transplantation markedly improved erectile performance (ICP/MAP 0.84 ± 0.14 vs. 0.38 ± 0.07 in controls), coincident with enhanced neovascularization and upregulation of endothelial markers (CD31, von Willebrand factor). Mechanistically, miR-126-laden exosomes from the modified cells appeared to drive these effects by elevating IRS1 and KLF10 expression in the recipient tissue [59]. Exosomes from lentivirus-transfected MSCs (Exo-145) carrying miR-145 demonstrated superior efficacy in treating aged rats with bilateral cavernous nerve injury (BCNI) than unmodified exosomes (Exo). Exo-145 treatment significantly improved erectile function, increased maximal intracavernosal pressure (ICP/MAP ratio), and markedly reduced apoptosis and fibrosis of CCSMCs by targeting TGFBR2 [60].

#### 4.6.3. Neurogenic Erectile Dysfunction

Neurogenic ED, frequently resulting from cavernous nerve injury (CNI) following radical prostatectomy, is characterized by cavernous smooth muscle apoptosis and subsequent fibrosis. Exosomal therapies heavily target neural repair and cellular preservation in this context. Adipose-derived stem cells (ADSC-Exos) improved erectile function in rats with BCNI in a study by Kim et al. [61]. Repeated exosome injections significantly improved erectile function, objectively displayed as an increased intracavernosal pressure (ICP)/mean arterial pressure (MAP) ratio. The treatment also enhanced smooth muscle-to-collagen ratios and elevated expression of α-smooth muscle actin, neuronal nitric oxide synthase, and cyclic guanosine monophosphate levels. RNA sequencing identified Ras homolog family member B as a predominantly expressed gene, promoting angiogenesis and endothelial cell proliferation [61]. To maximize these effects, engineered cargo has proven highly effective; for example, MSC exosomes enriched in miR-145 (or melatonin-pretreated exosomes yielding miR-145-5p) drastically reduce CCSMC apoptosis and fibrosis by inhibiting the TGFBR2 and TGF-β2/Smad3 pathways [62]. ADSC and bone marrow-derived (BMSC) exosomes have been shown to repair neurovascular markers (nNOS, neurofilament, vWF) and improve smooth muscle ratios following nerve injury [63]. Liu et al. [64] moved a step further and designed an injectable, thermosensitive hydrogel (HG) encapsulating adipose mesenchymal stem cell-derived exosomes (ADSC-Exo) to manage CNI-ED (cavernous nerve injury (CNI)-related erectile dysfunction) with enhanced therapeutic retention and slow release of the bioactive component at cavernous nerve injury (CNI) sites. In a rat model, HG-encapsulated ADSC-Exo significantly improved erectile function compared to unbound exosomes, demonstrating superior application features and structural stability [64]. A major translational hurdle in treating CNI-ED is the rapid washout of intratunical injections. To combat this, recent studies have successfully encapsulated ADSC exosomes within injectable, thermosensitive hydrogels (such as PELA loaded with polydopamine nanoparticles), ensuring sustained release over several weeks and yielding superior structural and functional recovery compared to unbound exosomes [65]. Alternative sources, such as low-intensity pulsed ultrasound (LIPUS)-stimulated Schwann cell exosomes, directly promote axon elongation and regeneration via the PI3K-Akt-FoxO pathway [66].

**Figure 8:**
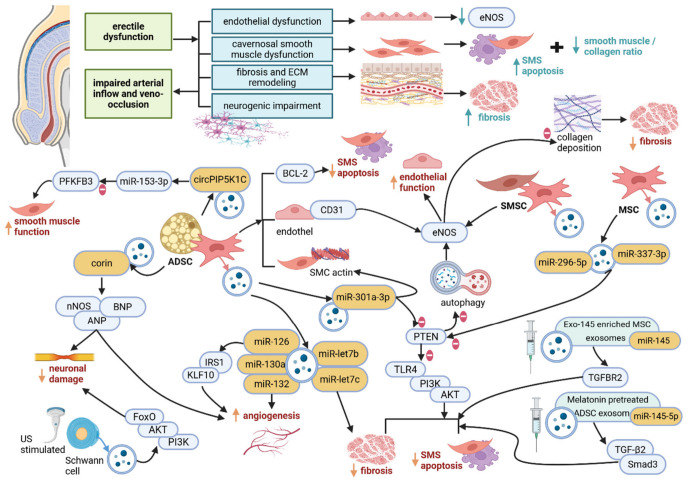
Pathophysiology of erectile dysfunction and stem/stromal cell-derived extracellular vesicle-mediated mechanisms of cavernosal repair. The upper panel depicts the multifactorial pathogenesis of erectile dysfunction, including endothelial dysfunction, cavernosal smooth muscle dysfunction, fibrosis with extracellular matrix remodeling, and neurogenic impairment. The lower panel illustrates regenerative and immunomodulatory mechanisms mediated by adipose-derived stem cells (ADSCs), mesenchymal stem cells (MSCs), and their extracellular vesicles/exosomes. ADSC-derived paracrine signals and microRNAs (e.g., circPIP5K1C–miR-153-3p–PFKFB3 axis) enhance smooth muscle function and suppress apoptosis through BCL-2 upregulation. Endothelial repair is promoted via increased CD31 expression and activation of eNOS, supported by cytoskeletal stabilization and enhanced autophagy. Pro-angiogenic effects are mediated by exosomal microRNAs (miR-126, miR-130a, miR-132, miR-let-7b/c) acting through IRS1/KLF10 signaling. Neuroprotective mechanisms include corin–ANP/BNP–nNOS signaling and ultrasound-stimulated Schwann cell activation via PI3K/AKT/FoxO pathways, reducing neuronal damage. Antifibrotic and pro-survival effects are achieved through suppression of TLR4/PTEN signaling and activation of PI3K/AKT, thereby reducing SMSC apoptosis and collagen accumulation. MSC-derived exosomes enriched in miR-145 or miR-145-5p, including melatonin-preconditioned ADSC exosomes, further inhibit TGF-β/TGFBR2/Smad3 signaling, attenuating fibrosis and promoting smooth muscle preservation. Together, these coordinated effects restore cavernosal structure and function, and improve erectile capacity (created with Biorender.com).

Ultimately, current preclinical studies position exosomes as a highly versatile, cell-free delivery system capable of intercepting multiple disease mechanisms in ED. The translation of these therapies to clinical practice will depend heavily on standardizing cargo engineering and optimizing local dosing strategies (Figure 8 and Table 9).

Erectile dysfunction is the benign urological condition closest to clinical testing, with several early-phase human trials now registered; nonetheless, the functional benefits reported to date (e.g., improvements in the intracavernous-pressure-to-mean-arterial-pressure ratio) originate from rodent models and cannot be directly extrapolated to patients.

### 4.7. Infertility

This section focuses on male factor infertility and andrology. Vesicles of the female reproductive tract are discussed only as mechanistic context for sperm maturation and function; female factor infertility is outside the scope of this review.

Infertility is a ubiquitous global problem, affecting approximately one out of six couples worldwide. Male factor infertility represents approximately 40–50% of all infertility cases, and it can occur due to different mechanisms, for example, damage to gametogenic cells, infection, and obstruction of the male reproductive tract, causing low sperm count and/or poor sperm quality [67]. Spermatogenesis is a complex process that relies on the balance of rigorous gene transcription and translation and stringently governed hormonal, temperature, and microenvironmental conditions. Sperm isolated from the testicle is still immotile, immature, and incapable of fertilizing an egg on its own, although it can be used for intracytoplasmic sperm injection. Sperm mature during transit through the epididymis, where morphologic, chemical, and other changes occur, giving them fertilization capacity. Part of this change is due to the transfer of RNAs, proteins, and other materials from the epididymis to sperm through extracellular vesicles [68]. The diagnostic evaluation of male infertility typically involves standard diagnostic features, including a detailed medical history, physical examination, and laboratory testing. The essential investigation is semen analysis (spermiogram), which reveals key information on sperm production and function. Additional diagnostics may include hormonal profiling (FSH, LH, testosterone, prolactin), genetic testing (e.g., karyotype, Y-chromosome microdeletions, CFTR mutations), scrotal or transrectal ultrasound to assess structural abnormalities, and specialized assays such as sperm DNA fragmentation testing or advanced imaging for sperm function. Spermiogram parameters consist of sperm volume, concentration, and number, pH, leucocyte count, motility, morphology, and vitality of spermatozoa. Abnormalities in these parameters can be categorized as oligozoospermia—low concentration; asthenozoospermia—reduced sperm motility; teratozoospermia—abnormal sperm morphology; or azoospermia—absence of sperm. The most pronounced abnormality in semen analysis helps guide further diagnostic workup and therapeutic decision-making. NOA is the most severe form of male infertility, defined as impaired or absent spermatogenesis within the testes. The usual etiological factors accounted for in primary testicular failure consist of genetic defects (e.g., Y-chromosome microdeletions, Klinefelter syndrome), prior gonadotoxic therapies, cryptorchidism, or idiopathic factors. In men with azoospermia, histological findings are often limited to a few recurring patterns, most commonly Sertoli cell-only syndrome, maturation arrest, or hypospermatogenesis. Non-obstructive azoospermia (NOA) remains particularly challenging in clinical practice. Spermatozoa are absent from the ejaculate, and retrieval is possible only in a proportion of patients, usually through testicular biopsy or microdissection (TESE). Obstructive azoospermia (OA) follows a different mechanism, with intact spermatogenesis, but impaired sperm transport. This reflects either congenital bilateral absence of the vas deferens linked to CFTR mutations, or acquired obstruction following infection, vasectomy, or some other cause of ductal scarring. Sperm retrieval rates are high in OA because spermatogenesis remains intact in these pathologies, and assisted reproductive techniques such as ICSI using epididymal or testicular sperm generally yield favorable outcomes [69].

Various methods have been used to restore fertility, including medications such as hormonal therapy, surgical therapy, and assisted reproductive technologies, but with limited results. Exosomes are a promising therapeutic effector in regenerative medicine, with substantial therapeutic potential in the management of infertility [67]. Exosomes are naturally produced by the male reproductive system and consist of epididymosomes and prostasomes. During epididymal transit, proteins harbored in epididymosomes are transferred to intracellular sperm domains, where they prompt the acquisition of fertilizing ability, modulate motility, and protect against oxidative stress. Prostasomal proteins also stimulate sperm motility, with an extra feature, which is the prevention of the premature induction of the acrosome reaction and protection of spermatozoa from immune responses within the female reproductive tract [70]. Collectively, exosome-associated proteins are indispensable for sperm maturation and represent valuable biomarkers for the early diagnosis of male infertility and potential targets for therapeutic exploitation [68].

Exosomes are increasingly recognized as important biomarkers in the evaluation of male infertility [71]. Their molecular cargo in seminal plasma correlates with sperm quality and reproductive potential. For example, PIWI-interacting RNAs (piRNAs) are significantly diminished in exosomes from men with asthenozoospermia. This finding correlates with impaired motility and diminished expression of the piRNA biogenesis factor MitoPLD [72]. Moreover, circular RNAs (circRNAs) isolated from seminal plasma exosomes of men with oligoasthenozoospermia display differential expression in nearly 15,000 transcripts, abundant in spermatogenesis-related pathways such as ubiquitin-mediated proteolysis, endocytosis, and RNA transport. These profiles suggest that exosomal circRNAs may serve as candidate diagnostic indicators of OA [73]. Other classes of exosomal RNAs have also shown clinical relevance. Transfer RNA-derived fragments (tRFs) in circulating plasma exosomes, particularly tRF-Gly-GCC-002 and tRF-Glu-CTC-005, have been identified as accurate predictors of successful sperm retrieval in men with non-obstructive azoospermia [74]. Similarly, seminal plasma small extracellular vesicle (sEV) miRNAs could differentiate between obstructive and non-obstructive azoospermia. Among them, miR-31-5p has shown more than 90% sensitivity and specificity, especially when combined with serum FSH levels, while additional miRNAs (miR-539-5p, miR-941) further better the prediction of residual spermatogenesis [75]. Differences in exosomal RNA and protein cargo are also evident when comparing fertile and infertile men. In oligoasthenozoospermic patients, increased levels of miR-765 and miR-1275 have been reported, alongside reduced expression of miR-15a and miR-34b-5p, when compared with normozoospermic controls [76,77]. At the protein level, seminal plasma exosomes from normozoospermic men are enriched in CRISP1, while those from asthenozoospermic patients contain inhibitory proteins such as glycodelin [78]. Likewise, prostasomes from infertile men display altered expression of proteins linked to sperm motility and energy metabolism [79]. At the same time, seminal plasma exosomes in varicocele-associated infertility show dysregulation of proteins such as ANXA2 (upregulated) and KIF5B (downregulated) [80].

At the post-translational level, altered citrullination and homocitrullination patterns (increase in homocitrulline (hCit) and a reduction in citrulline (Cit) modifications in proteins) in seminal plasma exosomal proteins further distinguish fertile from infertile men [81]. Additional exosome-based biomarkers have been described in diverse contexts. Exosomal RNA profiles from seminal plasma correlate with intrauterine insemination (IUI) outcomes, with successful pregnancies showing reduced total RNA and specific mRNA/lincRNA patterns, particularly increased expression associated with RNA originating from chromosomes 1, 10, 12, 16, and 21 [82]. In animal models, seminal plasma exosomes differentiate high- from low-motility sperm phenotypes through distinct proteomic signatures of proteins involved mainly in sperm–egg interaction, fertilization, and signaling pathways such as calcium and cAMP [83]. In contrast, lncRNA-miRNA-mRNA regulatory networks identified in asthenozoospermia exosomes suggest complex transcriptional dysregulation of genes predominantly involved in metabolism, transcription, ribosomal function, and channel activity [84]. Infectious conditions, such as orchitis or hepatitis B virus (HBV) infection, also influence exosomal cargo, altering the levels of important regulatory microRNAs, including miR-155-5p and miR-122-5p. These modifications are interconnected with testicular inflammation, impaired Sertoli cell metabolism, and an increased risk of infertility [85,86]. Overall, current evidence indicates that both seminal plasma and circulating exosomes carry disease- and fertility-specific molecular signatures. The RNA and protein content mirrors ongoing spermatogenesis and sperm function and has been associated with treatment outcomes, such as sperm retrieval rates and intrauterine insemination (IUI) success. Exosomes, therefore, represent a promising class of noninvasive biomarkers for the diagnosis, prognosis, and mechanistic understanding of male infertility [87]. Experimental in vitro work has shown that seminal plasma exosomes may also affect endometrial epithelial cells. Exosomes from men with oligoasthenoteratozoospermia are associated with lower expression of several transcripts involved in endometrial receptivity, including LIF, MUC1, G-CSF, CX3CL1, and VEGF. How these changes relate to clinical implantation outcomes remains an important question [88]. Seminal plasma-derived extracellular vesicles have also been examined in sperm cryopreservation. Their addition to freezing media has been linked to better post-thaw sperm characteristics and lower levels of oxidative stress-related damage. How these effects translate into assisted reproductive practice remains an area of ongoing investigation [89].

Exosomes have also been studied as noninvasive delivery systems for sperm biology research and therapeutic innovation. HEK293T-derived exosomes, when incubated with boar sperm, were rapidly internalized within 10 min, yet caused no detrimental effects on motility, viability, membrane integrity, or mitochondrial function even after several hours. Overall, the data indicates an acceptable safety profile and suggests that these vesicles could be used as carriers of molecular cargo in experimental and therapeutic applications [90]. In addition to male-derived vesicles, female reproductive tract exosomes also participate in sperm modifications and preservation and may establish a novel therapeutic avenue for improving fertility. Extracellular vesicles isolated from vaginal luminal fluid (vaginosomes) endorse sperm function by releasing proteins such as PMCA1, PMCA4, SPAM1, and tyrosine-phosphorylated proteins, which augment capacitation and acrosome reaction rates [91]. Similarly, sperm incubation with follicular fluid-derived exosomes enhanced progressive and total motility and evoked hyperactivation, which is a critical step for fertilization [92]. Exosomes also act as natural carriers of bioactive compounds, as illustrated after the exposure of prostatic cells to dietary lycopene, which resulted in the release of lycopene-enriched exosomes. The property of such vesicles is not only the protection of sperm from oxidative stress but also the contribution to the chemoprevention of prostate-related diseases, including benign prostatic hyperplasia (BPH) and prostate cancer, thereby linking exosomal transport to both male fertility and urological health [93]. Therapeutic applications of exosomes have also been demonstrated under conditions of testicular stress. When exposed to scrotal hyperthermia, both exosome therapy (EXO) and photobiomodulation therapy (PBMT) significantly improved sperm quality, preserved testicular architecture, and increased the number of germ cells, Leydig cells, and Sertoli cells, as well as testosterone levels. These treatments also reduced oxidative stress markers (ROS, oxidized glutathione [GSSG]) while elevating glutathione (GSH), which led to the protection of spermatogenesis from thermal injury [94].

Recent studies further emphasize the essential role of exosomes in pathophysiology, diagnosis, and treatment of non-obstructive azoospermia (NOA). Mainstream research focuses on Sertoli cell-derived exosomes, which mediate the transfer of regulatory microRNAs to spermatogonial stem cells (SSCs). Specifically, miR-486-5p delivered by Sertoli exosomes upregulates Stra8 and suppresses PTEN, subsequently promoting SSC differentiation. Inhibition of these exosomes results in the disruption of differentiation, implying that exosomal miR-486-5p is a potential therapeutic target in NOA [95]. Experimental models have also provided mechanistic insight into how exosomes regulate spermatogonial biology [96]. In essence, exosomes originating from Sertoli cells facilitate cellular communication essential for the proliferation of spermatogonial stem cells (SSCs) [97]. In vitro studies have demonstrated that Sertoli cell exosomes supply miR-30a-5p, which modulates the Zeb2/Fgf9 axis through the MAPK signaling pathway, consequently guiding spermatogonial stem cell (SSC) proliferation and differentiation. This elucidates the prospect of exosome-based approaches to enhance SSC regeneration [98]. Furthermore, a murine model-based experiment demonstrated that the RNA exosome-associated DIS3 ribonuclease is essential for spermatogonial stem cell homeostasis and germ cell differentiation [99]. In vivo, therapeutic application of urine-derived stem cell exosomes (USC-exos) restored spermatogenesis in busulfan-induced NOA mouse models. Subsequent to the treatment, spermatogenic genes (Pou5f1, Prm1, SYCP3, DAZL) and proteins, such as UCHL1, were upregulated, highlighting a promising, noninvasive therapeutic avenue [100]. Taken together, these studies demonstrate that exosomes elucidate mechanisms involved in the regulation of spermatogenesis and represent novel diagnostic and therapeutic tools for NOA.

In the context of obstructive azoospermia (OA), where sperm are absent from the ejaculate due to blockages in the reproductive tract, despite preserved spermatogenesis, exosomes represent both biomarkers and functional mediators of male fertility. In addition to their diagnostic potential, exosomes have been implicated in the sperm DNA integrity mechanism. Studies of spermatozoa from OA patients demonstrated that exosomes contain RNAs involved in DNA repair pathways. DNA repair genes PMS1, TP53BP1, and TLK2, which are associated with pathways such as the PI3K-Akt, p53 signaling, and cGMP-PKG pathways, are downregulated in sperm with a high DNA fragmentation index (DFI). These discoveries point to the contribution of exosomes to maintaining the genomic stability of sperm and place them as a potential diagnostic and therapeutic target for OA-related infertility [101]. Epididymal epithelial cells package key proteins into exosomes (“epididymosomes”) that fuse with transiting sperm and fine-tune their function. Wu and colleagues identified junctional adhesion molecule A (JAM-A) in epididymal vesicles and showed that it is transferred to sperm during epididymal transit [102]. In experimental settings, metabolic stress induced by a high-fat diet has been linked to endoplasmic reticulum stress and changes in epididymal exosome protein content, accompanied by impaired sperm development and fertility. The extent to which such mechanisms contribute to male infertility warrants further investigation [103].

**Figure 9:**
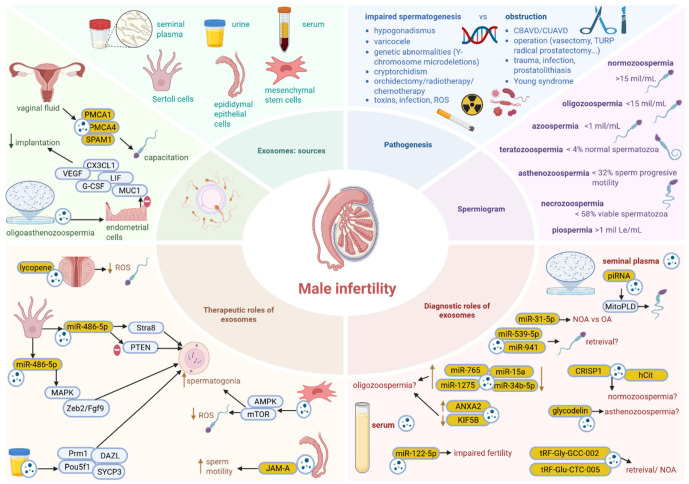
Exosome-mediated regulation of male infertility. This schematic illustrates the central role of exosomes in the pathophysiology, diagnosis, and treatment of male infertility. Exosomes derived from seminal plasma, urine, serum, and testicular somatic cells (including Sertoli cells, epididymal epithelial cells, and mesenchymal stem cells) act as key mediators of intercellular communication within the male reproductive tract. Through the transfer of bioactive cargo—such as microRNAs, piRNAs, proteins, and metabolic regulators—exosomes modulate spermatogenesis, sperm maturation, capacitation, oxidative stress responses, and sperm–female tract interactions. Pathogenetically, exosomal signaling influences both impaired spermatogenesis and obstructive etiologies by regulating germ cell differentiation, meiotic entry (e.g., STRA8), survival pathways (PTEN/PI3K–AKT, AMPK–mTOR), and inflammatory or toxic stress responses. Clinically, exosome-associated biomarkers detectable in seminal plasma or serum mirror distinct spermiogram phenotypes, including oligo-, astheno-, terato-, and azoospermia, and show promise for noninvasive discrimination between non-obstructive and obstructive azoospermia. Therapeutically, exosomes contribute to redox homeostasis, enhancement of sperm motility, and restoration of spermatogenic signaling, highlighting their potential as both biomarkers and biological effectors in male infertility (created with Biorender.com).

Amniotic fluid-derived exosomes (AF-Exo) and mesenchymal stem cell (MSC)-derived exosomes have both emerged as promising candidates for the treatment of male infertility. In experimental models of non-obstructive azoospermia, AF-Exos have been shown to significantly improve spermatogenesis and sperm quality. Treatment with these exosomes was associated with an increased number of OCT-3/4-positive progenitor cells and higher expression of spermatogonial markers, including DAZL and VASA. These changes are consistent with support of germ cell development [104]. MSC-derived exosomes act primarily through paracrine signaling. Bone marrow MSC-derived exosomes can modulate autophagy via the AMPK/mTOR pathway and are associated with reduced oxidative stress and inflammasome activity in the testes [105]. Adipose-derived MSC-derived exosomes improve testosterone production, enhance sperm motility, and upregulate important germ cell proteins, as demonstrated in models of chemotherapy-induced infertility [106]. hUCMSC-derived exosomes have been examined in busulfan-injured germ cells and mouse testes, where they are associated with changes in cell proliferation, migration, and spermatogenesis. Reduced oxidative stress and apoptosis have also been reported. The relevance of these observations for non-obstructive azoospermia remains under investigation [107]. Furthermore, mesenchymal stem cell (MSC)-derived exosomes have potential in treating sperm abnormalities and male infertility related to sexually transmitted diseases by reducing inflammation, cell damage, fibrosis, and scar formation [108]. Studies to date indicate that AF-Exos and MSC-derived exosomes may act as cell-free modulators of spermatogenesis and sperm function. Their molecular cargo provides a plausible basis for considering these vesicles in the development of less invasive interventions for male infertility (Figure 9 and Table 10).

### 4.8. Peyronie’s Disease

In Peyronie’s disease, a fibrotic disease of the tunica albuginea, exosomal therapy shows promise. Intratunical delivery of urine-derived stem cell exosomes (USC-exo) in a TGF-β1 rat model improved erectile mechanics (higher ICP/MAP) and restrained plaque formation, while suppressing fibroblast-to-myofibroblast transition and rebalancing matrix turnover by lowering TIMP-1/-2/-3 and boosting MMP-1, MMP-3 and MMP-9 activity. This antifibrotic profile supports disease pathogenesis, prevention and treatment [109].

Evidence in Peyronie’s disease is currently limited to a single preclinical study in a TGF-β1 rat model; the therapeutic potential of vesicles in this condition, although biologically plausible, should therefore be regarded as preliminary and requires confirmation in additional preclinical and, ultimately, clinical studies.

## 5. Synthesis, Challenges and Future Perspectives

### Clinical Translation, Regulatory Status, and the Current Trial Landscape

Despite encouraging preclinical results, the clinical translation of vesicle-based diagnostics and therapeutics in benign urology remains at an early stage. To date, no exosome- or EV-based product has received marketing approval from the U.S. Food and Drug Administration (FDA), the European Medicines Agency, or any comparable regulatory authority for any indication. The FDA has repeatedly stated that exosome products offered for therapeutic use are unapproved biologics, and has issued public safety notifications and warning letters following reports of serious adverse events—including infections and inflammatory reactions—associated with unapproved exosome preparations [110].

Human clinical experience specific to urological conditions is limited to a small number of early-phase and observational studies, most of which address erectile dysfunction. Registered examples include early-phase interventional trials of umbilical cord mesenchymal stem cell-derived exosomes (e.g., ClinicalTrials.gov NCT07319533 and NCT07480161) and platelet-rich plasma-derived exosomes (NCT07124871), together with observational studies of adipose-derived stem cell exosomes (NCT06605508) and regenerative approaches in gonadal dysfunction and azoospermia (NCT06841328). These studies are generally small, single-center, and, at the time of writing, either not yet recruiting or not yet completed; adequately powered, controlled efficacy data are therefore not yet available.

Several manufacturing and quality-control challenges must be addressed before vesicle-based therapies can enter routine practice. These include donor and cell-source variability, batch-to-batch heterogeneity, and the current absence of standardized potency assays and certified reference materials. Scalable, good manufacturing practice (GMP)-compliant production, comprehensive product characterization, and validated assays for sterility, endotoxin, and residual protein and lipoprotein content are all required. Biological uncertainties—including optimal dose, route of administration, biodistribution, rapid clearance, off-target uptake, and the potential presence of proinflammatory, profibrotic, or tumor-promoting cargo—further complicate translation. Accordingly, the disease-specific findings summarized in this review should be regarded as largely hypothesis-generating, and their clinical utility awaits confirmation in well-designed human studies.

Exosomes provide a unifying, mechanistically grounded framework across the urological disease spectrum. These selectively loaded, endosome-derived vesicles traffic RNAs, proteins, and lipids that reprogram recipient cells and thereby encode inflammation, matrix remodeling, neurovascular plasticity, immune set-points, and drug resistance [2,3,5,7]. In urology, this biology is unusually tractable because high-yield matrices (urine, semen, prostatic fluid) permit serial, low-burden access to exosomal cargo and, reciprocally, allow local delivery [2,6].

In urinary tract infections (UTIs), urothelial exosomes propagate danger signals. For instance, UPEC triggers urothelial pyroptosis and exosomal IL-1 and IL-18 release, recruiting mast cells and compromising the barrier [29]. Exosomes from infected epithelium drive macrophage TNF-α and apoptosis via miR-18a-5p, which triggers the PTEN/MAPK–JNK pathway. In contrast, inhibiting exosome release (GW4869) attenuates inflammation [30]. Host defense mechanisms are also mediated through exosome-based pathways. Urinary exosomes are enriched in innate immune proteins and lactoferrin, and exogenous lactoferrin reduces UPEC adherence and bacterial burden [31]. At the diagnostic interface, urinary exosomal Akt/CD9 patterns help discriminate UTI from asymptomatic bacteriuria, an area with frequent clinical ambiguity [32]. In IC/BPS, exosomes mirror and may drive neuroimmune activation (NK1R down-modulation; TLR4/NF-κB/NLRP3), while urinary lncRNA MEG3 emerges as a candidate biomarker [21,22,24]. Mesenchymal stromal cell exosomes (MSC-Exos) reduce mast cell infiltration, apoptosis, and fibrosis and restore pro-regenerative signaling, supporting intravesical, cell-free strategies [18].

In stress urinary incontinence, multiple preclinical models demonstrate consistent regenerative effects. Bone marrow-, adipose tissue-, and urine-derived stem cell-derived exosomes promote satellite cell proliferation and differentiation through ERK signaling. Moreover, these exosomal products preserve sphincter thickness, reduce fibrosis, and improve leak point pressure and bladder capacity [44,46,47,48]. The therapeutic effect of exosomes can be enhanced through engineering. Examples include SIRT1-overexpressing MSC-Exos and platelet-exosomal hydrogels, both improving local retention and therapeutic exposure [45,49,50].

For urethral stricture, where fibrosis is the central pathological process, macrophage- and stem cell-derived exosomes modulate core fibrogenic pathways. M2 macrophage-derived exosomes carrying M2 miR-34a-5p block autophagosome–lysosome fusion and promote fibrosis. Counter-programming via adipose-derived stem cell exosomes suppresses TGF-β/Smad and PDGFR-β/RAS/ERK, reducing collagen deposition and improving urodynamics [36,37]. Additionally, IL-1β-primed MSC-derived exosomes enriched in let-7c polarize macrophages to pro-resolving states and inhibit fibroblast activation, M2-derived exosomes containing miR-381 restrain YAP/GLS1-dependent glutaminolysis to limit myofibroblast transformation, and bone marrow MSC-derived exosomes downregulate Col I, fibronectin and elastin and upregulate pro-angiogenic factors [39,40,41]. Tissue engineering expands these possibilities. Bioactive nanoyarn scaffolds, augmented with ADSC-Exos, promote epithelialization and vascularization while minimizing scarring [38]. These findings, taken together, suggest that antifibrotic, exosome-based methods could be useful supplementary treatments for urethral strictures, alongside endoluminal and reconstructive procedures [34].

Extracellular vesicle signaling also contributes to male reproductive physiology and infertility. Epididymosomes and prostasomes are physiologic vesicles that deliver proteins and small RNAs required for sperm maturation, motility, capacitation, and immune tolerance in the female tract [68,70]. Their dysregulation provides noninvasive windows into spermatogenesis. For example, a drop in seminal plasma exosomal piRNAs occurs in asthenozoospermia [72], while circRNA and tRF cargo discriminate between oligozoospermia and asthenozoospermia and predict micro-TESE success in NOA [73,74,75]. Proteomic and post-translational signatures, to be more precise, glycodelin enrichment, altered citrullination/homocitrullination and ANXA2/KIF5B in varicocele, differentiate fertile from infertile men [79,80,81]. Exosomes also capture inflammatory and viral influences. UPEC orchitis and HBV infection remodel Sertoli and testicular exosomal miR-155-5p/miR-122-5p with consequences for metabolism and immunity [85,86]. Functionally, Sertoli cell exosomes govern SSC proliferation and differentiation through pathways involving miR-486-5p, miR-30a-5p/Zeb2/Fgf9 axis and DIS3 dependence. Furthermore, urine- or umbilical cord-derived MSC-derived exosomes restore spermatogenesis in NOA models [95,98,99,100,105,107]. Additional vesicle systems, including vaginosomes and follicular fluid exosomes, modulate capacitation and motility, while benign HEK293T-derived exosomes interact rapidly with sperm without impairing function, supporting safe delivery concepts [90,91,92]. Together, these findings argue for embedding seminal or urinary exosomal panels alongside semen analysis and hormones in andrology workflows, and for progressing paracrine exosomal therapies toward translation [67,71].

Finally, in terms of erectile dysfunction, across diabetic, hypoxic, arterio-injury, and nerve injury models, ADSC-, BMSC-, and Schwann-cell-derived exosomes restore endothelial and smooth muscle integrity, raise eNOS/nNOS, suppress apoptosis and fibrosis, and improve functional erectile parameters such as intracavernosal pressure relative to mean arterial pressure [51,52,53,58]. Engineering exosomal cargo with delivering molecules such as corin, miR-145, miR-296-5p/miR-337-3p, miR-301a-3p and circPIP5K1C enhances angiogenesis, autophagy, and antifibrosis [54,55,56,57]. Thermosensitive hydrogels extend cavernosal dwell time and boost efficacy [64,65]. Translationally, erectile dysfunction is a rational early target for local, cell-free therapy with objective vascular and patient-reported endpoints [61,62,63,111].

Exosomes give urology a single, mechanistic scaffold spanning diverse urological benign diseases. In cystitis/IC, they mediate the transfer of host defense signals and neuroimmune mediators, and can be leveraged for intravesical, cell-free therapy [18,24,31]. In incontinence and urethral fibrosis, they orchestrate myofascial repair and antifibrotic reprogramming, enabling minimally invasive, regenerative approaches [36,37,38,44,46]. In infertility, they encode sperm maturation states and spermatogonial stem cell dynamics while offering predictive and restorative avenues [71,72,73,95,100]. In erectile dysfunction, they restore neurovascular function with measurable hemodynamic improvements and maturing delivery platforms [51,52,64].

Overall, the field is advancing, supported by growing interdisciplinary collaboration between clinicians, translational scientists, and bioengineers; nonetheless, clinical translation in benign urology remains at an early and largely preclinical stage, and enthusiasm should be tempered by the regulatory and manufacturing hurdles outlined above. Such efforts will be essential to refine current methodologies, strengthen the evidence base, and enable the safe and effective incorporation of exosome-based technologies into clinical practice.

## Figures and Tables

**Table 1:** A comparison between stem cell therapy and stem cell-derived exosome therapy.

	Adult Stem Cell Therapy	Stem Cell-Derived Exosome Therapy
**Therapeutic Potential**	Broad, due to multilineage differentiation	Comparable benefits via paracrine signaling
**Scientific Evidence**	Extensive preclinical and clinical data	Increasing preclinical data; clinical trials emerging
**Cell Source & Production**	Easy to isolate; scalable	Low yield; challenging large-scale production
**Regulatory Framework**	Well-established	Underdeveloped; lacks global standardization
**Storage & Transport**	Requires strict conditions (cryopreservation)	Stable; suitable for long-term storage
**Viability Post Delivery**	Limited cell survival and engraftment	Stable and functional after administration
**Risk of Tumorigenicity**	Present, especially in pluripotent cells	Reduced relative to cell therapy, but not fully excluded
**Immunogenicity**	Potential immune response	Generally low, but context-dependent and not eliminated
**Ethical Concerns**	Absent	Fewer than for cell-based therapy
**Delivery Routes**	Often invasive (e.g., injections)	Multiple options (intravenous, topical, local)
**Cargo & Function**	Relies on live cell activity	Can be engineered with specific proteins, miRNAs, etc.
**Batch Consistency**	Variable; donor-dependent	More uniform but depends on production method
**Toxicity Risk**	Possible due to cell behavior in vivo	Lower, but not yet fully characterized
**Current Limitations**	Ethical issues, storage, tumor risk, toxicity	Low yield, lack of purification/storage protocols

**Table 2:** Comparison between extracellular vesicles.

Extracellular Vesicle Type	Size	Formation Mechanism	Clinical Relevance
**Exosomes**	40–160 nm	Formed via the endocytic pathway	Most widely utilized in tissue repair and regenerative medicine (especially MSC-derived exosomes)
**Ectosomes (e.g., microvesicles, microparticles and large vesicles)**	50–1000 nm	Produced by outward blebbing of the plasma membrane and released following proteolytic cleavage of the cytoskeleton	Less commonly utilized compared to exosomes
**Apoptotic bodies**	500–2000 nm	Generated as by-products of programmed cell death	Least utilized among extracellular vesicles in regenerative medicine

**Table 3:** EV isolation methods.

Method	Principle	Advantages	Disadvantages	Suitable For
**Ultracentrifugation (UC)**	Size/density-based sedimentation	Widely used Label-free Scalable	Time-consuming High cost Low purity Potential structural damage	Large-volume samples
**Density Gradient Centrifugation**	Sedimentation through sucrose/iodixanol gradient	Higher purity than UC alone	Time-consuming (viscous media—slow sedimentation)	Purification after UC
**Polymer Precipitation (PEG)**	Reduces solubility to precipitate exosomes	Easy Inexpensive High throughput	Low purity Possible false positives Hard-to-remove polymers	Virus-like particles Fast screening
**Size-Exclusion Chromatography (SEC)**	Separation by size via porous matrix	Gentle on vesicles Preserves structure Low cost	May co-isolate similarly sized particles	Biofluids (serum, urine) Downstream assays
**Ultrafiltration**	Filtration through membranes with a specific MWCO	Rapid Simple No need for special equipment	Low purity Nonspecific binding Membrane clogging	Pre-concentration Assisting other methods
**Immunoaffinity Capture (IAC)**	Antibody-based selection of surface markers	High specificity/purity Small volume needed Quantification possible	Costly Complex Not suitable for large-scale use	Targeted exosome populations Diagnostics

**Table 4:** EV characterization and quantification assays.

Assay	Min. Detectable Vesicle Size (nm)	Advantages	Limitations
**Nanoparticle Tracking Analysis (NTA)**	70–90	Precise size and concentration measurement Detects biological markers Highly sensitive	Limited resolution in multimodal samples Risk of overestimation Needs optimal dilution
**Transmission Electron Microscopy (TEM)**	~1	High resolution Visual confirmation	Multi-step preparation Technically demanding
**Immune Capture-Based ELISA (IC-ELISA)**	Not size-dependent	Specific, sensitive, reproducible Low sample volume Cost-effective	Requires pre-enrichment (ultracentrifugation) Target-specific only

**Table 5:** Preservation and storage techniques.

Method	Principle	Advantages	Disadvantages	Suitable For
**Cryopreservation**	Storage at low temperatures (4 °C, −80 °C, −196 °C)	Preserves function short-term; widely available	Ice crystal damage; requires antifreezes; expensive for long-term	Short-term storage; lab-scale preservation
**Freeze-Drying**	Freeze + vacuum sublimation of ice	Room temp storage; long shelf-life; preserves activity with additives	Needs cryoprotectants (e.g., trehalose); possible structural damage	Long-term storage; clinical and transport use
**Spray-Drying**	Atomization in hot air to remove moisture	Fast; economical; scalable; adjustable particle size	Sensitive to temperature/pressure; potential damage to EV structure	Industrial processing; oral drug delivery
**−80 °C** **Freezing**	Direct low-temp freezing	Simple and common method	Alters morphology and reduces bioactivity over time (after ~28 days)	Short-term lab storage
**With Antifreezes**	Use of permeable (DMSO) or non-permeable (trehalose)	Prevents ice damage; maintains structure and function	Requires optimization of concentration; some toxicity concerns	Enhancing other storage methods (e.g., freezing)

**Table 6:** Level of evidence for extracellular vesicle applications across benign urological disorders. Most disease-specific findings are preclinical; human data are confined to exploratory biomarker studies and early-phase erectile dysfunction trials.

Condition	Highest Current Evidence	Representative Model/Sample	Application	Clinical Readiness
**IC/BPS**	Human biomarker (exploratory) + preclinical therapy	Human urine (MEG3); rat and feline models	Diagnostic + therapeutic	Preclinical; biomarker exploratory
**UTI**	Mechanistic (in vitro/animal) + human biomarker	UPEC-infected urothelium; human urine	Mechanistic + diagnostic	Preclinical/early diagnostic
**Neurogenic bladder**	Human biomarker	Human urine (vitronectin)	Diagnostic	Exploratory biomarker; no therapy
**Urethral stricture**	Preclinical	Rat, rabbit, urethral fibroblasts	Therapeutic (antifibrotic)	Preclinical
**Stress urinary incontinence**	Preclinical	Rat, porcine, fibroblasts	Therapeutic (regenerative)	Preclinical
**Erectile dysfunction**	Preclinical + early-phase human trials	Rat models; phase 1/2 trials	Therapeutic	Earliest clinical (trials ongoing)
**Male infertility**	Human biomarker + preclinical therapy	Seminal plasma; rat/mouse	Diagnostic + therapeutic	Preclinical; biomarker exploratory
**Peyronie’s disease**	Preclinical (single study)	TGF-β1 rat model	Therapeutic (antifibrotic)	Preliminary/preclinical

**Table 7:** Exosome-mediated regulation of fibrosis and urethral stricture: molecular cargo, signaling pathways, and experimental models.

Exosome Source	Key Molecule	Target/Pathway	Mechanism of Action	Observed Effects	Model	Ref
**Adipose-derived stem cells (ADSCs)**	miRNAs (unspecified)	TGF-β/Smad & PDGFR-β/RAS/ERK pathways	Suppression of fibrosis signaling via TGF-β pathway inhibition	Reduced collagen deposition, fibrosis reduction	Rat	[37]
**Adipose-derived stem cells (ADSCs)**	Exosomes (unspecified)	Inflammatory response, collagen synthesis	Promotes epithelialization and angiogenesis; inhibits fibroblast over-proliferation	Improved urethral healing, minimized fibrosis	Rat, human fibroblasts (in vitro)	[38]
**IL-1β-treated MSCs**	let-7c	PAK1/NF-κB	Induces M2 macrophage polarization; suppresses fibroblast activation	Reduced collagen synthesis and fibrosis	Rabbit, THP-1 monocytes (in vitro)	[39]
**Bone marrow MSCs (BMSCs)**	Exosomes (unspecified)	Fibrosis genes (Col I, fibronectin, elastin); angiogenesis genes (VEGF, eNOS, bFGF)	Suppression of fibrosis and stimulation of angiogenesis pathways	Prevention of fibrosis, reduced urethral stricture	Rat	[41]
**TNF-α-treated MSCs (human umbilical cord-derived)**	miR-146a	Inflammatory signaling pathways	Suppresses fibroblast activation and inflammation via anti-inflammatory miRNA delivery	Reduced fibrosis and urethral stricture	Rat, fibroblasts (in vitro)	[42]
**M2 macrophages**	miR-34a-5p	SIRT1/autophagy flux	Blocks autophagosome–lysosome fusion; increases fibroblast fibrogenesis	Enhanced fibrosis development	Rat, urethral fibroblasts (in vitro)	[36]
**M2 macrophages**	miR-381	YAP/GLS1/glutaminolysis	Suppresses glutaminolysis via YAP/GLS1 pathway inhibition	Reduced fibroblast activation and fibrosis	Rabbit, urethral fibroblasts (in vitro)	[40]

All available data in urethral stricture are preclinical (rodent, rabbit, and in vitro); no clinical studies have been conducted.

**Table 8:** Exosome-based regenerative therapies for urinary sphincter dysfunction: sources, molecular mediators, and mechanisms.

Exosome Source	Key Molecules/Markers	Mechanism/Pathway	Therapeutic Effects	Ref
**Platelet-Derived Extracellular Vesicle Product (PEP)**	CD41a, CD9, NF-κB, PD-L1	NF-κB signaling	Promotes muscle regeneration, urethral sphincter function, M2 macrophage polarization	[49]
**M2 Macrophage-Derived Exosomes (M2-EXO)**	miR-501	Targets YY1 gene	Enhances myoblast differentiation, reduces inflammation, accelerates muscle recovery	[50]
**SIRT1-Overexpressing Bone Marrow MSC-Derived Exosomes (SIRT1/exos)**	SIRT1	ERK signaling pathway	Improves urodynamics (ALPP, MBV), promotes satellite cell proliferation and differentiation	[44]
**Human Adipose-Derived Stem Cell-Derived Exosomes (hADSC-Exo)**	Proteins in PI3K-Akt, Jak-STAT, Wnt pathways	Muscle and nerve regeneration signaling pathways	Improves bladder capacity and leak point pressure, increases muscle and nerve fiber density	[46]
**Urine-Derived Stem Cell-Derived Exosomes (USCs-Exo)**	ERK-related proteins	ERK phosphorylation	Activates muscle satellite cells, improves muscle regeneration	[48]
**Adipose-Derived Mesenchymal Stem Cell-Derived Exosomes (adMSC-Exos)**	col1a1, TIMP-1, TIMP-3, MMP-1, MMP-2	Modulates collagen synthesis and degradation	Enhances collagen synthesis, reduces collagen degradation	[47]

**Table 9:** Exosome-based therapeutic strategies in experimental models of erectile dysfunction: sources, molecular mediators, mechanisms, and functional outcomes.

Study Model/Type of ED	Exosome Source	Administration Route	Key Molecular Mediators	Mechanism/Pathway	Main Outcomes	Ref
**Diabetes-induced ED**	Corpus cavernosum smooth muscle cells (CCSMC-EXOs)	Intracavernous injection	**eNOS, nNOS, NO, cGMP**	NO/cGMP signaling, antifibrosis	↑ erectile function, ↓ fibrosis, ↑ smooth muscle	[51]
**Diabetes-induced ED**	Adipose-derived stem cell (ADSC)-derived exosomes	Intracavernous injection	**CD31, α-SMA, Bcl-2, Cleaved Caspase-3**	Anti-apoptotic, endothelial markers	↑ erectile function, ↓ apoptosis, ↑ endothelial recovery	[52]
**Diabetes-induced ED**	ADSC-derived exosomes	Intracavernous injection	**miR-126, miR-130a, miR-132, let-7b/c**	Angiogenesis, antifibrosis	↑ erectile function, ↓ fibrosis, ↑ angiogenesis	[53]
**Diabetes-induced ED**	ADSC-derived exosomes (**corin protein**)	Intravenous injection	**Corin, ANP, BNP, nNOS**	Neurovascular, anti-inflammatory pathway	↑ erectile function, ↓ inflammation	[54]
**Age-related ED**	Mesenchymal stem cells (MSC-Exos)	Intracavernous injection	**miR-296-5p, miR-337-3p** (target PTEN)	PTEN/PI3K/AKT pathway	↑ erectile function, ↓ apoptosis	[55]
**Obstructive sleep apnea (OSA)–ED**	ADSC exosomes enriched in **miR-301a-3p**	Intracavernous injection	**miR-301a-3p (targets PTEN, TLR4)**	PTEN/HIF-1α and TLR4 pathway	↑ erectile function, ↓ apoptosis, ↑ autophagy	[56]
**OSA-induced ED**	ADSC exosomes containing **circPIP5K1C**	Intracavernous injection	**circPIP5K1C, miR-153-3p, SMURF1, PFKFB3**	Glycolysis inhibition pathway	↑ erectile function, ↓ apoptosis, smooth muscle recovery	[57]
**Arterial injury-induced ED**	MSC-derived exosomes	Intracavernous injection	**NO synthase (NOS)**, oxidative stress markers	Endothelial repair, oxidative stress reduction	↑ erectile function, ↓ oxidative stress	[58]
**Cavernous injury-induced ED**	Muscle-derived stem cells transfected with **miR-126**	Local transplantation	**miR-126 (targets IRS1, KLF10)**	Angiogenesis, antifibrotic effects	↑ erectile function, ↓ fibrosis, ↑ angiogenesis	[59]
**Neurogenic ED (BCNI)**	Bone marrow MSC-derived exosomes modified with **miR-145 (Exo-145)**	Intracavernous injection	**miR-145 (targets TGFBR2)**	TGFBR2 pathway	↑ erectile function, ↓ fibrosis, ↓ apoptosis	[60]
**Neurogenic ED (BCNI)**	MSC-derived exosomes	Intracavernous injection (repeat)	**Ras homolog family member B (RHOB)**	Angiogenesis pathway	↑ erectile function, neuronal/endothelial recovery	[61]
**Neurogenic ED (BCNI)**	Melatonin-pretreated ADSC exosomes (MT-hASC-EVs)	Intracavernous injection	**miR-145-5p (targets TGF-β2/Smad3)**	TGF-β2/Smad3 signaling	↑ erectile function, ↓ fibrosis	[62]
**Post-radical prostatectomy ED**	ADSC/BMSC-derived exosomes	Intracavernous injection	**nNOS, neurofilament,** **α** **-SMA, vWF**	Neurovascular regeneration	↑ erectile function, ↑ nerve regeneration, ↓ fibrosis	[63]
**Neurogenic ED (BCNI)**	ADSC exosomes in thermosensitive hydrogel (HG@Exo)	Intratunical injection	**Not specifically identified**	Sustained release system	↑ erectile function, ↓ fibrosis, ↑ exosome retention	[64]
**Vascular injury-induced ED**	ADSC exosomes in polydopamine nanoparticle hydrogel (**PDNPs-PELA**)	Intratunical injection	**Not specifically identified**	Sustained release, neuronal/endothelial repair	↑ erectile function, ↓ neuronal/endothelial damage	[65]
**Cavernous nerve injury-induced ED (CN-ED)**	Low-intensity ultrasound-stimulated Schwann cell exosomes (**LIPUS-SCs-Exo**)	Local administration	**miRNAs regulating PI3K-Akt-FoxO pathway**	PI3K-Akt-FoxO signaling	↑ erectile function, ↑ nerve regeneration	[66]

**Table 10:** Sources, molecular cargo, and functional roles of exosomes in male reproductive physiology, fertility, and infertility.

Exosomes	Function	Study Type	Molecule	Reference
**Epididymal exosomes (epididymosomes)**	Transfer proteins to sperm, enabling maturation, motility acquisition and protection from oxidative stress	Physiological/molecular studies	Proteins transferred to sperm intracellular domains; JAM-A	[70,102]
**Prostatosomes**	Enhance sperm motility, prevent premature acrosome reaction and protect sperm from immune responses within the female reproductive tract	Functional and proteomic studies	Prostasomal proteins related to motility and metabolism	[70,79]
**Seminal plasma exosomes**	Biomarkers of sperm quality, infertility diagnosis and reproductive potential; regulate sperm maturation, motility, DNA stability and fertilization processes; influence endometrial receptivity and sperm cryopreservation outcomes	Clinical biomarker studies, proteomics, transcriptomics and experimental studies	piRNAs, circRNAs, tRF-Gly-GCC-002, tRF-Glu-CTC-005, miR-31-5p, miR-539-5p, miR-941, miR-765, miR-1275, miR-15a, miR-34b-5p, CRISP1, glycodelin, ANXA2, KIF5B, citrullination/homocitrullination modifications, LIF, MUC1, G-CSF, CX3CL1, VEGF, DNA repair genes (PMS1, TP53BP1, TLK2)	[71,72,73,74,75,76,77,78,80,81,82,83,87,88,89,101]
**Circulating plasma exosomes**	Predict successful sperm retrieval in non-obstructive azoospermia	Clinical biomarker study	tRF-Gly-GCC-002, tRF-Glu-CTC-005	[74]
**Infection-associated exosomes**	Reflect inflammatory responses and impaired spermatogenesis during infections	Clinical molecular studies	miR-155-5p, miR-122-5p	[85,86]
**Female reproductive tract exosomes (vaginosomes and follicular fluid EVs)**	Promote sperm capacitation, acrosome reaction, motility and fertilization potential	Experimental studies	PMCA1, PMCA4, SPAM1, tyrosine-phosphorylated proteins	[91,92]
**Sertoli cell-derived exosomes**	Regulate spermatogonial stem cell proliferation and differentiation; mediate germ cell communication in spermatogenesis	In vitro studies	miR-486-5p, miR-30a-5p; Stra8, PTEN; Zeb2/Fgf9 axis	[95,96,97,98]
**RNA exosome complex**	Maintains spermatogonial stem cell homeostasis and germ cell differentiation	Animal model study	DIS3 ribonuclease	[99]
**Stem cell-derived exosomes (USC, MSC, AF-Exo)**	Restore spermatogenesis, improve sperm quality, reduce oxidative stress, and regulate autophagy and inflammation	Experimental animal models and regenerative studies	Pou5f1, Prm1, SYCP3, DAZL, OCT-3/4, VASA, germ cell proteins; AMPK/mTOR signaling	[100,104,105,106,107,108]
**Engineered or experimental exosomes**	Potential delivery systems for therapeutic molecules targeting sperm biology	In vitro experimental study	Exosomal molecular cargo	[90]
**Prostate-derived lycopene exosomes**	Protect sperm from oxidative stress and contribute to chemoprevention of prostate diseases	Experimental study	Lycopene-enriched exosomes	[93]
**Epididymal exosomes under metabolic stress**	Alter sperm development and fertility under metabolic conditions such as high-fat diet	Animal study	ER-stress-associated proteins	[103]
**Therapeutic exosomes in hyperthermia models**	Improve spermatogenesis and reduce oxidative stress during testicular heat stress	Animal study	ROS ↓, GSH ↑, GSSG ↓	[94]

In male infertility, seminal-plasma vesicle studies provide human biomarker data, whereas therapeutic evidence (e.g., amniotic fluid- and MSC-derived vesicles in azoospermia) remains confined to animal models.

## Data Availability

No new data were created or analysed in this study. Data sharing is not applicable to this article.
